# CRC-TrustBench: benchmarking cross-study and cross-country generalization in gut metagenomic colorectal cancer prediction

**DOI:** 10.3389/fmicb.2026.1971707

**Published:** 2026-09-25

**Authors:** Yuzheng Zhang, Yangzong Cidan, Jiayi Xu, Bingtong Li

**Affiliations:** 1 Department of Hospital Infection Management, China-Japan Union Hospital of Jilin University, Changchun, China; 2 Department of Epidemiology and Biostatistics, School of Public Health, Jilin University, Changchun, China; 3 Department of Scientific Research, China-Japan Union Hospital of Jilin University, Changchun, China; 4 Department of Rheumatology and Immunology, China-Japan Union Hospital of Jilin University, Changchun, China

**Keywords:** colorectal cancer, cross-cohort generalization, data leakage, gut metagenomics, uncertainty-aware prediction

## Abstract

**Background:**

Gut metagenomic classifiers for colorectal cancer (CRC) are often evaluated with random splits that place samples from the same studies, countries, or processing workflows in both training and test sets, potentially inflating apparent performance. We developed CRC-TrustBench, a leakage-aware framework for evaluating cross-study and cross-country generalization and probability reliability.

**Methods:**

From 2,340 MetaGenoPolis V16 source samples, we curated 1,953 CRC or healthy samples from 15 studies and 10 countries; 185 adenoma samples were retained outside binary model development as a challenge population. Subject-aware random, leave-one-study-out (LOSO), and leave-one-country-out (LOCO) protocols were applied to taxonomic profiles with 2,543 features and functional profiles with 378 features. We compared Elastic Net, LightGBM, cohort-balanced Elastic Net, and pre-specified neural domain-robust methods using fold-specific pre-processing. The Microbiome Leakage and Confounding Audit (MiLCA) quantified study and country shortcuts, while Platt calibration, conformal-style sets, and selective abstention were evaluated without outer-test labels.

**Results:**

For taxonomic profiles, LightGBM achieved an area under the receiver operating characteristic curve (AUROC) of 0.874 under random splitting, 0.827 under LOSO, and 0.821 under LOCO; the single pre-specified RANDOM estimate was numerically higher than the held-out-domain summaries. Microbiome profiles predicted study and country identity, and a descriptive feature-level audit indicated pervasive study-associated structure. Cohort balancing yielded limited gains, and neural domain-robust methods did not consistently outperform LightGBM. Platt scaling reduced the mean LOCO Brier score from 0.180 to 0.169 and log loss from 0.630 to 0.521. LightGBM conformal-style coverage fell below the pre-specified 90 and 95% targets, while uncertainty-based abstention reduced error relative to matched-random rejection in most recorded repeats. Functional profiles were less predictive, although more functional features met the pre-specified high-stability tiers.

**Discussion:**

The tiered features remain stable candidate hypotheses, and raw counts are not directly comparable across the differently sized feature spaces. Adenoma predictions were heterogeneous, and no reviewed external resource met all criteria for formal independent evaluation. CRC-TrustBench separates in-distribution discrimination from cohort generalization and probability reliability without implying clinical deployment or independent external validation.

## 1 Introduction

Colorectal cancer (CRC) remains a major cause of cancer morbidity and mortality, and prevention depends on detecting neoplasia before symptoms appear. Fecal shotgun metagenomics offers a non-invasive view of the gut microbial community at both taxonomic and functional levels. Individual cohorts have reported discriminative taxonomic patterns, and meta-analyses have identified signals that recur across countries and study designs (Zeller et al., 2014; Yu et al., 2017; Feng et al., 2015; Dai et al., 2018; Yachida et al., 2019; Wirbel et al., 2019; Thomas et al., 2019). Gut metagenomic profiles are therefore useful for studying disease associations and their reproducibility, but these observations alone do not establish a screening test. Relationships between the microbiome and colorectal tumorigenesis are heterogeneous, and observational profiles also reflect host, environmental, clinical, and technical influences (Tilg et al., 2018; Wong and Yu, 2019).

Samples collected within one study often share recruitment pathways, geography, specimen handling, sequencing procedures, and bioinformatic processing. Random assignment across training and test sets can preserve this cohort-specific information on both sides of a split, so a realized in-distribution estimate can exceed held-out-domain estimates without establishing transportability. Although previous CRC metagenomic studies have examined cross-cohort discrimination, few have considered cohort shortcuts, held-out-country performance, probability calibration, conformal uncertainty, and selective abstention in a common leakage-controlled analysis. A useful benchmark must ask more than whether CRC and healthy samples can be ranked: it should establish what domain information the profiles encode, how performance changes when an entire study or country is unseen, whether the resulting probabilities remain reliable, and where the evidence ends.

CRC-TrustBench addresses these questions through four linked analyses. We first curated an international public metagenomic release into subject-aware CRC-vs.-healthy and adenoma populations governed by immutable split and matrix policies. We then compared subject-aware random splitting with leave-one-study-out (LOSO) and leave-one-country-out (LOCO) evaluation while measuring how strongly microbial profiles encoded study and country identity. Conventional, cohort-balanced, and pre-specified domain-robust models were followed by probability calibration, conformal-style set construction, and selective abstention, with outer-test labels excluded from model selection, calibration, and threshold estimation. Secondary taxonomic and functional stability analyses were kept separate from descriptive adenoma prediction and qualification of candidate external resources.

Domain-generalization algorithms do not consistently outperform carefully implemented empirical baselines when model selection and validation are controlled (Gulrajani and Lopez-Paz, 2021; Zhou et al., 2023). We therefore retained negative, non-significant, and non-evaluable findings. CRC-TrustBench provides an auditable evaluation of cross-study and cross-country generalization, cohort shortcuts, probability reliability, and the generation of stable candidate hypotheses. Its scope does not extend to independent external validation, clinical deployment, causal microbial mechanisms, or replacement of established CRC screening.

## 2 Related work

### 2.1 Gut metagenomic CRC prediction and cross-cohort generalization

Public microbiome collections are particularly vulnerable to optimistic evaluation. Samples are clustered by study, recruitment pathway, geography, storage, extraction, sequencing, and annotation, yet conventional random splitting can distribute samples from the same clusters across training and test sets. A classifier may then exploit cohort-associated features even when nominal sample identifiers do not overlap. Laboratory and analytical choices produce measurable variation in microbial profiles, while medication, demographic characteristics, and other host variables can confound disease associations (Costea et al., 2017; Sinha et al., 2017; McLaren et al., 2019; Vujkovic-Cvijin et al., 2020; Forslund et al., 2015). This is not a defect unique to one dataset but a general property of multi-study observational microbiome data.

Reproducible microbiome prediction consequently requires explicit subject grouping, fold-local pre-processing, a declared feature namespace, and validation at the level where distribution shift is expected (Knight et al., 2018; Schloss, 2018; Wang and LêCao, 2020; Wirbel et al., 2021). LOSO and LOCO protocols cannot substitute for a new prospective cohort, but they address a question that random splitting leaves unresolved: how much discrimination remains when an entire recorded study or country is absent from model development? Earlier CRC meta-analyses demonstrated that part of the microbial signal is shared across cohorts, while also revealing substantial variation in transportability (Wirbel et al., 2019; Thomas et al., 2019). Relative to these studies and general microbiome meta-analysis toolkits, CRC-TrustBench contributes a fixed integration of identity governance, fold-local processing, country-label holdout, cohort-structure audits, and probability-reliability analyses rather than a new classifier. Cross-domain performance should therefore be treated as a distinct estimand rather than inferred from an in-distribution test set.

Compositionality adds another layer of sensitivity. Relative-abundance profiles occupy a constrained sample space, and analytical results can change with zero handling, normalization, filtering, and differential-abundance procedures (Aitchison, 1986; Gloor et al., 2017; Quinn et al., 2019; McMurdie and Holmes, 2014; Nearing et al., 2022). Any sample-dependent pre-processing operation must be estimated within the current training fold to prevent information from validation or test samples from influencing the representation. Taxonomic and functional profiles are both conditional on reference databases and annotation workflows; functional profiles nevertheless provide a useful secondary view of shared microbial capacity (Truong et al., 2015; Beghini et al., 2021; Franzosa et al., 2019; Kanehisa and Goto, 2000).

We therefore treated taxonomic and functional analyses as separate estimands. We also distinguished predictive discrimination from biological stability. A feature may contribute to classification without showing a consistent cross-study association, whereas a modestly predictive functional signal may remain directionally stable across cohorts. Model coefficients, feature importance, meta-analytic consistency, and experimental validation answer different questions and should not be collapsed into a single biomarker claim.

### 2.2 Reliable machine learning under cohort shift

Domain-robust learning attempts to reduce performance degradation when training and test distributions differ. Simple approaches include study-balanced sampling or loss weighting, whereas representation-learning approaches attempt to align domain distributions or optimize performance for poorly performing groups. Deep CORAL reduces differences between representation covariances across training domains, while GroupDRO emphasizes groups with higher training risk (Sun and Saenko, 2016; Sagawa et al., 2020). However, large comparative studies have shown that specialized domain-generalization objectives do not consistently outperform well-tuned empirical-risk-minimization baselines when model selection is controlled (Gulrajani and Lopez-Paz, 2021; Koh et al., 2021; Zhou et al., 2023). Domain-robust methods should therefore be evaluated against strong conventional models under identical pre-processing, split, and selection contracts.

A related problem is determining whether the input data encode domain identity strongly enough to support shortcut learning. Predicting study or country from microbiome profiles does not prove that a CRC classifier uses the same information, but high domain predictability establishes that such information is available. Comparisons among diagnosis-only, metadata-only, missingness-only, and microbiome-only audit models can help distinguish label imbalance from broader cohort structure. Feature-level comparisons of study-associated and diagnosis-associated effects provide an additional descriptive view, although they cannot identify the causal source of the domain signal. Such analyses should be treated as audit instruments rather than as deployable disease predictors or *post hoc* feature-removal rules.

Discrimination alone is also an incomplete account of model behavior. The area under the receiver operating characteristic curve (AUROC) is insensitive to the numerical reliability of predicted probabilities, and a model with useful ranking may remain systematically overconfident or underconfident after a domain change. Calibration methods assess and adjust the probability map (Niculescu-Mizil and Caruana, 2005; Guo et al., 2017). Proper scoring rules such as the Brier score and log loss evaluate probability quality, whereas calibration error summarizes agreement between predicted and observed frequencies.

Split conformal prediction can convert calibration scores into set-valued outputs with finite-sample coverage guarantees under exchangeability assumptions (Vovk et al., 2005; Angelopoulos and Bates, 2023). However, exchangeability is precisely what cross-study or cross-country shift can threaten. Coverage measured on held-out domains should therefore be interpreted empirically rather than presented as a universal guarantee for future populations.

Selective classification addresses a related operational question by evaluating whether abstaining on samples with high predictive uncertainty yields a retained subset with fewer errors than an equally sized subset produced by random rejection (El-Yaniv and Wiener, 2010; Geifman and El-Yaniv, 2017). Dataset-shift studies demonstrate that uncertainty estimates themselves can deteriorate when the test distribution changes (Ovadia et al., 2019). Calibration, conformal prediction, and selective abstention should therefore accompany, rather than replace, discrimination analysis. Together, they characterize different aspects of reliability while remaining distinct from evidence of clinical utility.

## 3 CRC-TrustBench design and evaluation protocol

CRC-TrustBench is a leakage-aware benchmark for colorectal cancer (CRC) prediction from gut metagenomic profiles. It combines version-controlled data curation, subject-level identity reconciliation, fold-specific feature processing, multilevel generalization assessment, cohort-shortcut analysis, and uncertainty-aware prediction (Figure 1). The primary task evaluated CRC-vs.-healthy discrimination. Adenoma samples were kept outside binary model development and used as a separate descriptive challenge population. Subject-aware random splitting provided an in-distribution reference, while LOSO and LOCO validation assessed cross-study and cross-country generalization, respectively.

**Figure 1:**
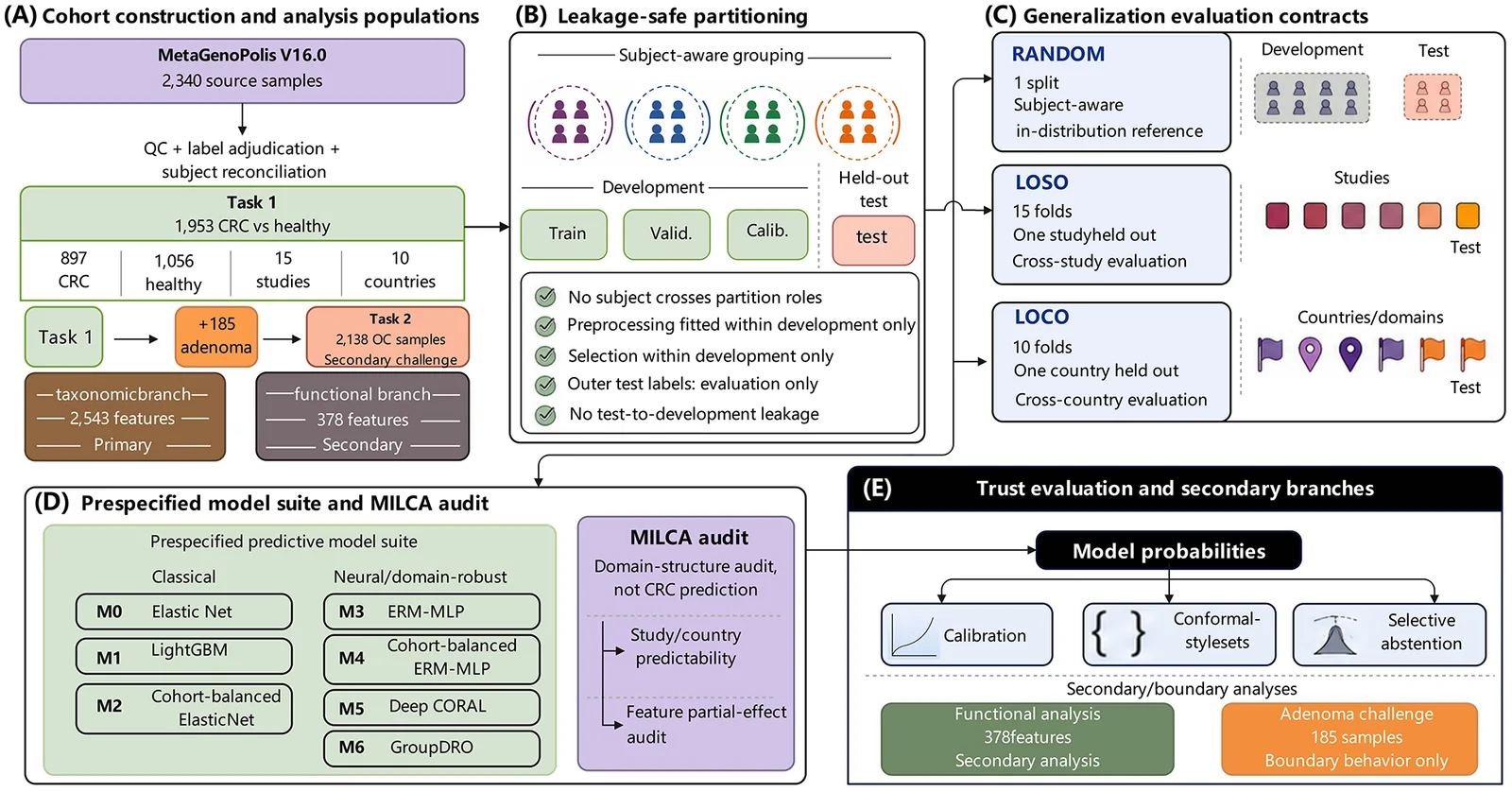
CRC-TrustBench study design and analysis flow. **(A)** Cohort construction and analysis populations. **(B)** Subject grouping and fold-local leakage control. **(C)** The single pre-specified subject-aware RANDOM split, leave-one-study-out (LOSO), and leave-one-country-out (LOCO) evaluation. **(D)** Pre-specified predictive models and the Microbiome Leakage and Confounding Audit (MiLCA). **(E)** Probability calibration, conformal-style prediction sets, selective abstention, and secondary analyses. No reviewed external resource met all pre-specified requirements for formal independent evaluation.

Throughout, information leakage denotes prohibited transfer of subject identity or held-out-partition information into training, selection, or pre-processing. Cohort-shared structure is distinct: studies or countries may occur on both sides of an otherwise subject-disjoint RANDOM split, defining an in-distribution reference that can expose potential shortcut information without constituting identity leakage. Confounding denotes association of domain factors with the outcome or features, whereas domain shift denotes a change in distributions across partitions. The Microbiome Leakage and Confounding Audit (MiLCA) tested whether domain information was available in the profiles, not whether a CRC classifier used it.

### 3.1 Data source, curation, and analysis populations

We performed a retrospective secondary analysis of publicly available human fecal shotgun-metagenomic data. The source was MetaGenoPolis V16.0 (Barbet et al., 2022). The release comprised 2,340 samples from 15 studies and 10 countries and included manually curated metadata, taxonomic profiles, and functional profiles. Dataset metadata, file identifiers, retrieval dates, official checksums, and local SHA-256 values were registered before analysis. Source archives were retained separately from working matrices and derived results.

Eligibility required human fecal material, shotgun metagenomic sequencing, a stable sample identifier, an identifiable study and country, a resolvable diagnostic category, and an available profile permitted by the matrix allowlist. The original metadata fields were retained, and diagnostic labels were mapped to the canonical categories CRC, healthy, adenoma, other disease, or unknown under a version-controlled harmonization policy. Diagnoses were not inferred from study titles, cohort names, or coarse health-status fields. In particular, 27 PRJEB6070 samples supported by the source metadata, archive records, and publication evidence as small adenomas remained classified as adenoma and were not collapsed into the healthy group.

The diagnosis mapping retained explicit source class labels before harmonization. Diagnostic confirmation, disease stage, and treatment status were not available in the canonical records, whereas age and sex availability varied by study. The availability audit did not impute unavailable fields, and no additional age-, sex-, stage-, or treatment-adjusted models were fitted (Supplementary Table S15).

Quality-control decisions preceded model development. Samples with sample-level evidence of contamination or failure to meet the source-defined high-quality clean-read threshold were excluded from the primary modeling population but retained in the adjudication record. The primary sequencing-depth policy excluded samples marked as having fewer than five million high-quality clean reads. Records with fewer than one million reads defined a sensitivity boundary for severely low-depth samples and did not replace the sample-level source flags. Documentary inconsistencies were recorded as limitations; sample status was not revised after model results became available.

Repeated samples from the same individual were reconciled before fixed split construction. For the longitudinal PRJDB4176 records, the pre-surgical time point (*t1*) was used in the primary analysis, whereas the post-surgical time point (*t2*) was excluded from primary model fitting and retained only for documented longitudinal sensitivity interpretation. Raw identifiers appearing in more than one study were namespaced by study and were not considered to represent the same participant without accession-level evidence.

The resulting primary population, Task 1, contained 1,953 subject-grouped samples: 897 CRC and 1,056 healthy. The quality-controlled three-category population, Task 2, contained 2,138 samples after inclusion of 185 adenoma samples. Task 2 defined the adenoma challenge and descriptive reference distributions. Adenoma samples did not contribute to binary model fitting, hyperparameter selection, probability calibration, conformal quantiles, or selective-abstention thresholds. Figure 1 summarizes the population flow and analytical roles.

### 3.2 Leakage control and fold-specific feature processing

Canonical sample, subject, and subject-group identifiers were propagated through the population files, split assignments, prediction records, and result tables. All observations associated with the same subject group were constrained to one analytical partition. Cross-study identifiers were namespaced before metadata or matrix joins. Potential duplicate profiles were retained for explicit adjudication rather than automatically interpreted as repeated participants.

Pre-defined matrix allowlists and blocklists governed model inputs. The primary taxonomic input was an uncorrected source matrix containing 2,543 metagenomic species-pangenome features, and the secondary input contained 378 functional features. Twenty-eight release-derived matrices had undergone global correction, filtering, or transformation incompatible with fold-specific estimation; these were blocked by both file path and content hash. The modeling pipeline rejected a matrix if either identifier matched the blocklist.

No global batch correction was applied before validation. Within every RANDOM, LOSO, and LOCO fold, prevalence filtering, zero replacement, centered log-ratio (CLR) transformation, scaling, and model-specific feature selection were estimated using the corresponding training partition only. Candidate prevalence thresholds were pre-specified and evaluated only within the development data. The zero-replacement parameter was estimated from the training samples and then applied unchanged to validation, calibration, and test samples. CLR transformation followed the compositional-data rationale of Aitchison and related microbiome guidance (Aitchison, 1986; Gloor et al., 2017). Scaling parameters were likewise learned only from the training partition.

Taxonomic identifiers were retained in their source namespace. Matrix transposition was treated as a deterministic orientation operation and did not involve sample-dependent estimation. The functional workflow followed the same leakage-control principles as the taxonomic workflow. Taxonomic and functional representations were evaluated separately and were not combined into a multimodal predictor.

Study identifiers, country labels, demographic variables, protocol fields, and missingness indicators were excluded from the primary microbiome feature matrices and used only in the planned audit analyses. Outcome labels were not reused as domain labels, and domain identifiers were never supplied as predictive features to the CRC classifiers. Figure 1 shows the matrix-governance and fold-specific processing workflow.

### 3.3 Benchmark tasks and fixed validation protocols

Task 1 evaluated binary discrimination between CRC and healthy samples, with taxonomic and functional profiles analyzed as separate feature views. Task 2 retained the quality-controlled adenoma samples as a descriptive challenge using models frozen after Task 1. Stable taxonomic and functional candidate hypotheses were generated only after completion of the predictive benchmark and were not fed back into model fitting or feature selection.

The adenoma challenge applied the frozen Task 1 models to the 185 quality-controlled adenoma samples without refitting. Because adenoma was not assigned to either class in the binary prediction task, conventional binary accuracy was not calculated. Instead, calibrated CRC probabilities, conformal-style prediction sets, uncertainty scores, selective-retention status, and training-distribution distances were summarized descriptively and compared with the internal healthy and CRC reference distributions. Adenoma samples did not contribute to model development, probability calibration, conformal-quantile estimation, selective-abstention thresholds, or distribution-shift thresholds.

We used three fixed validation protocols. The primary RANDOM evaluation used a single pre-specified subject-group split into training, validation, calibration, and test partitions while allowing samples from the same studies and countries to appear in different analytical roles. No subject group crossed partitions. RANDOM therefore served as an in-distribution reference and was not treated as the principal estimate of generalization to unseen cohorts. A sensitivity analysis repeated the same subject-aware RANDOM procedure across 30 fixed split seeds for M0 and M1 while holding the population, role proportions, stratification, fold-local pre-processing, validation-only tuning, and model seed fixed. It quantified split-to-split variability without replacing the primary RANDOM protocol; no RANDOM-vs.-LOSO or RANDOM-vs.-LOCO significance test was performed (Supplementary Table S9).

The LOSO protocol held out one complete study in each outer fold and served as the primary cross-study evaluation. Fifteen study folds were defined. Two held-out studies contained only one outcome class; metrics requiring both classes, including AUROC and the area under the precision–recall curve (AUPRC), were therefore recorded as not estimable and excluded from macro discrimination means while remaining visible in fold counts and probability-based summaries.

The LOCO protocol held out one complete country in each outer fold and assessed cross-country generalization. Ten country folds were defined. Because some study records spanned more than one country, country holdout did not guarantee simultaneous exclusion of every contributing study. LOCO should therefore be interpreted as a country-label holdout within the release rather than a crossed study-and-country design or independent external validation (Supplementary Table S10).

Within each outer fold, pre-processing and candidate models were fitted using the training partition, and the validation partition was used for bounded hyperparameter and model selection. After model selection, the final uncalibrated model was fitted according to the frozen protocol using the permitted development data while leaving the calibration partition untouched. The calibration partition was reserved for probability calibration, conformal-quantile estimation, selective-abstention thresholds, and distribution-shift thresholds. The outer test partition was transformed and evaluated only after all fitting and selection procedures had been completed. Under LOSO and LOCO, the held-out study or country was excluded from all fitting, pre-processing, model-selection, calibration, and threshold-estimation steps.

Split files were versioned and linked to hashes of the population manifest, eligibility policy, and matrix-governance files. Automated checks verified that subject groups were disjoint across training, validation, calibration, and test partitions; every included sample had exactly one analytical role in each fold; and the held-out study or country was absent from all fitting roles. Figure 1 illustrates the RANDOM, LOSO, and LOCO designs.

External-resource evaluation was treated as a separate qualification task rather than as an extension of the development data. Candidate resources were required to contain human fecal shotgun-metagenomic profiles, interpretable sample-level labels, stable accession identifiers, licensing compatible with analysis, and processed features that could be mapped to the frozen internal feature space. Independence was adjudicated using project identifiers, run and BioSample accessions, sample-level overlap, BioProject membership, publication linkage, licensing, and feature compatibility. Resources sharing the same BioProject, accession, or biological sample with the development data were not considered independent external evaluations, whereas the same samples processed through a different analytical pipeline were classified as pipeline replications. Only resources with explicit authorization, adequate sample-level identity information, and compatible processed feature profiles were eligible for application of the frozen models. External data were not used to modify the internal feature space, fit pre-processing parameters, tune models, recalibrate probabilities, or select conformal, abstention, or distribution-shift thresholds.

### 3.4 Predictive models and domain-robust learning

Three conventional models formed the principal comparison. M0 was Elastic Net logistic regression, M1 was LightGBM, and M2 was cohort-balanced Elastic Net (Friedman et al., 2010; Ke et al., 2017). Elastic Net provided a sparse linear reference, whereas LightGBM represented a non-linear tree-based learner. Dummy prior and seeded stratified classifiers were included as non-informative references but were not candidates for the primary predictive model.

For M2, sample weights were calculated within each training partition so that every training study contributed the same total weight, with equal weighting of samples within each study. The held-out study or country did not contribute to weight calculation. M2 used the same feature-processing and validation contract as M0, thereby isolating the effect of cohort balancing from changes in model class or feature representation.

Hyperparameters were selected from compact, pre-specified grids using the internal validation data. The selection criterion prioritized macro performance across validation studies rather than sample-weighted performance alone. Threshold-dependent metrics used a fixed probability threshold of 0.5 in the principal analysis; no outer-test labels were used to optimize a classification threshold.

Four neural models were included as supplementary domain-robust comparisons. M3 was a standard empirical-risk-minimization multilayer perceptron (ERM-MLP), M4 was a cohort-balanced ERM-MLP, M5 implemented Deep CORAL, and M6 implemented GroupDRO. The neural methods shared a compact feed-forward architecture with two hidden layers, layer normalization, Gaussian error linear unit (GELU) activations, and dropout. Batch normalization was not used because batch-level statistics could inadvertently encode cohort composition.

M4 used study-balanced sampling or equivalent study-balanced loss weighting within the training data. Deep CORAL added a covariance-alignment penalty to the classification loss to reduce representation differences among training studies (Sun and Saenko, 2016). GroupDRO updated study-level loss weights to emphasize training groups with higher current risk (Sagawa et al., 2020). Study identity was used only as a training-domain label for the relevant robustness objective and was never included in the feature vector presented to the classifier.

Neural analyses used the pre-specified random seeds 17, 29, and 43. Hyperparameter and epoch selection relied on grouped internal validation in which complete studies, rather than individual samples, defined validation groups. Early stopping did not use the outer test domain. Neural predictions were aggregated across seeds within each held-out domain before study-level comparisons. Figure 1 shows the predictive-model branch.

### 3.5 MiLCA cohort-shortcut and distribution-shift audits

MiLCA characterized domain information and potential shortcuts in the curated data. Its models served only as diagnostic instruments and were not used for CRC prediction.

Study and country predictability were assessed using pre-specified feature views: diagnosis alone, metadata missingness alone, available demographic metadata, diagnosis combined with metadata, and microbiome profiles alone. The microbiome-only audit used the same fold-specific prevalence, zero-handling, CLR, and scaling principles as the primary benchmark. Sample and subject identifiers were never included as predictors.

Multiclass study and country prediction was evaluated using subject-aware cross-validation with the pre-specified seeds 17, 29, and 43. The principal audit metric was macro-F1, supplemented by balanced accuracy, top-1 and top-3 accuracy, multiclass log loss, class-specific recall, and one-vs.-rest AUROC where estimable. Diagnosis-balanced training weights and repeated study-by-diagnosis down-sampling were used as sensitivity analyses to assess whether domain predictability could be explained solely by differences in CRC prevalence.

Metadata values and missingness indicators were evaluated separately. Numerical metadata were imputed and scaled using training-fold parameters only, and categorical variables were encoded without access to the test fold. Missingness indicators were restricted to the metadata audit and were not included in the primary microbiome models.

Feature-level domain structure was quantified for the 1,953 Task 1 subject groups and 2,543 taxonomic features using nested least-squares designs: intercept only, diagnosis alone, study alone, and diagnosis plus study. Study was a 15-level factor encoded by 14 indicators, whereas diagnosis was one binary indicator. For each taxonomic feature, partial effects were calculated from residual sums of squares (RSS) as


$$R_{\text{partial}}^{2}=\frac{\text{RS}{\text{S}}_{\text{reduced}}-\text{RS}{\text{S}}_{\text{full}}}{\text{RS}{\text{S}}_{\text{reduced}}}.$$

The full model included an intercept, diagnosis, and study. For the diagnosis term, the reduced model included an intercept and study; for the study term, it included an intercept and diagnosis. Thus, the denominator was the reduced-model RSS. Diagnosis-associated effects were adjusted for study and study-associated effects for diagnosis; age, sex, body mass index, treatment, stage, platform, and other clinical covariates were not included, and no missing-value imputation was applied. The descriptive comparison criterion was $R_{\text{study}}^{2}>R_{\text{diagnosis}}^{2}$. No feature-wise significance tests or *p* values were generated, so no multiplicity correction was applied. Because the study and diagnosis terms differ substantially in dimensionality and parameterization, this associational comparison was not complexity-adjusted and does not identify contamination, laboratory bias, geography, or another causal source.

For each LOSO fold, we summarized separation between the development data and the held-out study using an Aitchison-centroid distance after training-fold compositional transformation, principal-component-space distances, regularized Mahalanobis distances, and nearest-neighbor distances. Dimensionality reduction and covariance estimation used only the development data. Study-level Spearman correlations, bootstrap confidence intervals, and permutation tests related domain-distance measures and study predictability to held-out-study discrimination. Given the limited number of evaluable studies, these analyses were exploratory rather than confirmatory and were not intended to support causal inference. Figure 1 displays MiLCA separately from CRC prediction.

### 3.6 Calibration, conformal-style set construction, and selective abstention

Probability calibration and uncertainty analyses were performed within the fixed LOCO framework. For each held-out country, Platt scaling was fitted using the reserved internal calibration partition and applied unchanged to the outer-test predictions (Niculescu-Mizil and Caruana, 2005; Guo et al., 2017). Calibration was evaluated using the Brier score, log loss, calibration slope, and expected calibration error with at most 15 equal-frequency bins. Small held-out folds therefore used singleton bins, so their expected calibration error was treated as descriptive rather than as a conventional fixed-bin estimate. A legacy supplementary field originally labeled calibration intercept equals the outer-test prevalence log odds by construction and is not interpreted as a jointly fitted recalibration intercept. Isotonic regression was included as a sensitivity analysis only when the calibration partition satisfied the pre-specified minimum total and class-specific sample sizes.

Empirical conformal-style set construction used the calibrated class probabilities (Vovk et al., 2005; Angelopoulos and Bates, 2023). For calibration sample *i*, the non-conformity score was defined as


$$\begin{array}{l} s_{i}=1-{\hat{p}}_{i,y_{i}}, \end{array}$$ (1)

where ${\hat{p}}_{i,y_{i}}$ was the calibrated probability assigned to the observed class. The scores in Equation 1 were used with the finite-sample conformal quantile rule at α = 0.10 and α = 0.05, corresponding to nominal coverage levels of 90 and 95%. The resulting prediction sets could contain the healthy class, the CRC class, both classes, or neither class. We reported empirical marginal coverage, mean prediction-set size, singleton rate, ambiguous-set rate, and empty-set rate.

The same calibration records were used both to fit the Platt map and to estimate the conformal score quantiles. The predictor was therefore not fixed independently of the score-calibration data, so the usual split-conformal finite-sample coverage guarantee does not directly apply to these sets. Country-level domain shift may further violate exchangeability. Coverage was therefore interpreted as empirical behavior under the recorded LOCO protocol rather than as a distribution-free guarantee for future populations.

As a supplementary robustness analysis, we deterministically divided the frozen calibration role into subject-disjoint, class-stratified Calibration-A and Calibration-B subsets. Calibration-A was used to fit the Platt map, whereas Calibration-B supplied the conformal scores and quantiles; the held-out country remained evaluation-only. Primary calibration roles contained 140–212 records per held-out country and model, while Calibration-A and Calibration-B each contained 70–106 records (Supplementary Table S12). Selective abstention used predictive entropy as the primary uncertainty score (El-Yaniv and Wiener, 2010; Geifman and El-Yaniv, 2017). Thresholds corresponding to target coverages of 90% and 80% were determined using the calibration partition and applied unchanged to the held-out country. We reported realized test coverage, selective error, selective balanced error, rejection rate, retained CRC and healthy sample counts, class-specific retention rates, and country-specific behavior. Selective AUROC and AUPRC were calculated only when both outcome classes remained in the accepted subset.

To determine whether uncertainty-based abstention provided information beyond removing samples alone, each selective rule was compared with 200 fixed-seed random rejection replicates matched to the same number of accepted samples. We reported the difference between the uncertainty-based selective error and the mean random-rejection error, together with the proportion of random replicates for which uncertainty-based abstention produced lower error.

Sample-level training-distribution distances provided a descriptive adjunct. Principal-component and regularized Mahalanobis representations were fitted using the development data, and the calibration partition determined the pre-specified 95th- and 99th-percentile distance thresholds. These values were shift scores, not ground-truth out-of-distribution labels or causal explanations of prediction errors. Figure 1 summarizes calibration, conformal-style set construction, and selective abstention.

### 3.7 Statistical analysis and stable-hypothesis generation

The observation unit was a subject-grouped sample, whereas the principal generalization units were held-out studies and countries. Sample-level metrics were calculated to describe operating characteristics, but principal summaries used domain-level macro estimates so that the largest cohort did not determine the estimand by itself.

Discrimination was assessed using AUROC and AUPRC, with CRC treated as the positive class. Threshold-dependent metrics included balanced accuracy, sensitivity, specificity, and F1 score at the fixed threshold of 0.5. Probability reliability was assessed using the Brier score, log loss, and expected calibration error. AUROC was calculated only when both outcome classes were present. Single-class studies or countries remained in the analysis record; metrics requiring both outcome classes were reported as NA (not estimable) for these folds rather than assigned an artificial value.

Domain-level summaries included arithmetic macro means across evaluable domains, medians, worst-domain and second-worst-domain values, interquartile ranges, and between-domain standard deviations (SDs). Sample-weighted summaries, where reported, were kept separate from domain-macro summaries. For neural models, seed-level results were first aggregated within each held-out domain before comparisons were made between methods.

Exploratory between-domain uncertainty was summarized using 10,000 percentile bootstrap resamples, with the held-out study as the LOSO resampling unit and the held-out country as the LOCO resampling unit. These intervals describe variability among the observed domains and are neither prediction intervals for future domains nor clinical confidence intervals (Supplementary Table S14).

Methods were compared within the same held-out study or country. Paired differences were summarized using 10,000 paired bootstrap resamples and 10,000 sign-flip permutations; Wilcoxon signed-rank tests were reported as an additional non-parametric comparison. Multiple comparisons within each pre-defined family were controlled using the Benjamini–Hochberg (BH) procedure (Benjamini and Hochberg, 1995). Adjusted *p* values are reported as *q* values. Numerical gains were not described as statistically supported improvements when they did not remain supported after multiplicity correction. Correlations between domain-shift measures and model performance were considered exploratory because of the limited number of evaluable domains.

Cross-cohort taxonomic and functional hypotheses were generated independently of model selection. Within each eligible study, CRC-vs.-healthy effects were calculated on the CLR scale using mean differences, standardized mean differences, and Hedges' *g*. Study-specific effects were combined using random-effects meta-analysis. Heterogeneity was summarized using Cochran's *Q*, τ^2^, and the pre-specified *I*^2^ convention (Higgins and Thompson, 2002). Direction consistency was defined as the proportion of evaluable studies whose effect direction agreed with the pooled direction.

Leave-one-study-out influence analysis repeatedly recalculated the pooled effect after omitting one study and recorded direction reversals and influential cohorts. Elastic Net stability selection was performed within the outer LOSO development data using subject-group bootstrap resampling; the held-out study did not contribute to pre-processing, coefficient estimation, or selection-frequency calculation. LightGBM permutation importance was calculated using held-out predictions and changes in log loss or Brier score, without feeding importance values back into model fitting.

Candidate tiers were defined before the stability results were inspected. Meta-analysis *p* values were adjusted using the Benjamini–Hochberg procedure to obtain *q* values. Tier A required *q* < 0.05, direction consistency of at least 0.80, no leave-one-study-out direction reversal, Elastic Net selection frequency of at least 0.50, at least eight evaluable studies, *I*^2^ ≤ 50%, and no evidence that the effect was driven by one study. Tier B required *q* < 0.05, direction consistency of at least 0.70, Elastic Net selection frequency of at least 0.30, at least six evaluable studies, and *I*^2^ ≤ 75%. Tier C contained exploratory model-associated features that did not meet Tier A or B but satisfied at least two pre-specified criteria involving Elastic Net selection frequency, LightGBM permutation-importance rank, direction consistency, or diagnosis-associated incremental explained variance. These tiers were interpreted as stable candidate hypotheses rather than validated biomarkers or causal mechanisms. Multivariable association analyses followed established meta-omics practice (Mallick et al., 2021).

## 4 Results

### 4.1 Data curation established a leakage-safe analysis population

The MetaGenoPolis V16.0 source release contained 2,340 samples. After quality control, label adjudication, and subject-level reconciliation, Task 1 comprised 1,953 subject-grouped samples: 897 CRC and 1,056 healthy samples from 15 studies and 10 countries. Adding 185 adenoma samples produced the quality-controlled Task 2 population of 2,138 samples. The taxonomic and functional representations contained 2,543 and 378 features, respectively; 28 globally corrected or filtered matrices remained blocklisted. Source class labels supported diagnosis harmonization, but diagnostic confirmation, stage, and treatment were unavailable in the canonical records; age data were complete for 13 studies and unavailable for two studies, while sex data were complete for 12 studies, partial for two studies, and unavailable for one study (Supplementary Table S15). Table 1 and Figure 2 summarize cohort composition, and Supplementary Table S1 summarizes cohort composition and quality-control exclusions; the underlying population records are provided with the machine-readable Supplementary material.

**Table 1:** Cohort composition of the pre-specified analysis populations.

Study	Country	Task 1 (*n*)	CRC	Healthy	Adenoma	QC eligible (*n*)
PRJDB4176	JPN	547	257	290	67	614
PRJEB10878	CHN	123	70	53	0	123
PRJEB12449	USA	102	50	52	0	102
PRJEB27928	DEU	79	21	58	0	79
PRJEB6070	Multiple	152	87	65	37	189
PRJEB7774	AUT	97	34	63	46	143
PRJNA389927	Multiple	30	15	15	9	39
PRJNA397112	IND	74	0	74	0	74
PRJNA429097	CHN	189	96	93	0	189
PRJNA447983	ITA	105	57	48	26	131
PRJNA531273	IND	11	11	0	0	11
PRJNA608088	CHN	18	6	12	0	18
PRJNA731589	CHN	144	68	76	0	144
PRJNA763023	CHN	192	95	97	0	192
PRJNA961076	BRA	90	30	60	0	90
**Total**	–	**1,953**	**897**	**1,056**	**185**	**2,138**

Task 1 included colorectal cancer (CRC) and healthy samples; adenoma samples were retained only in the Task 2 challenge population. “Multiple” denotes studies containing samples from more than one country. QC denotes quality control.

**Figure 2:**
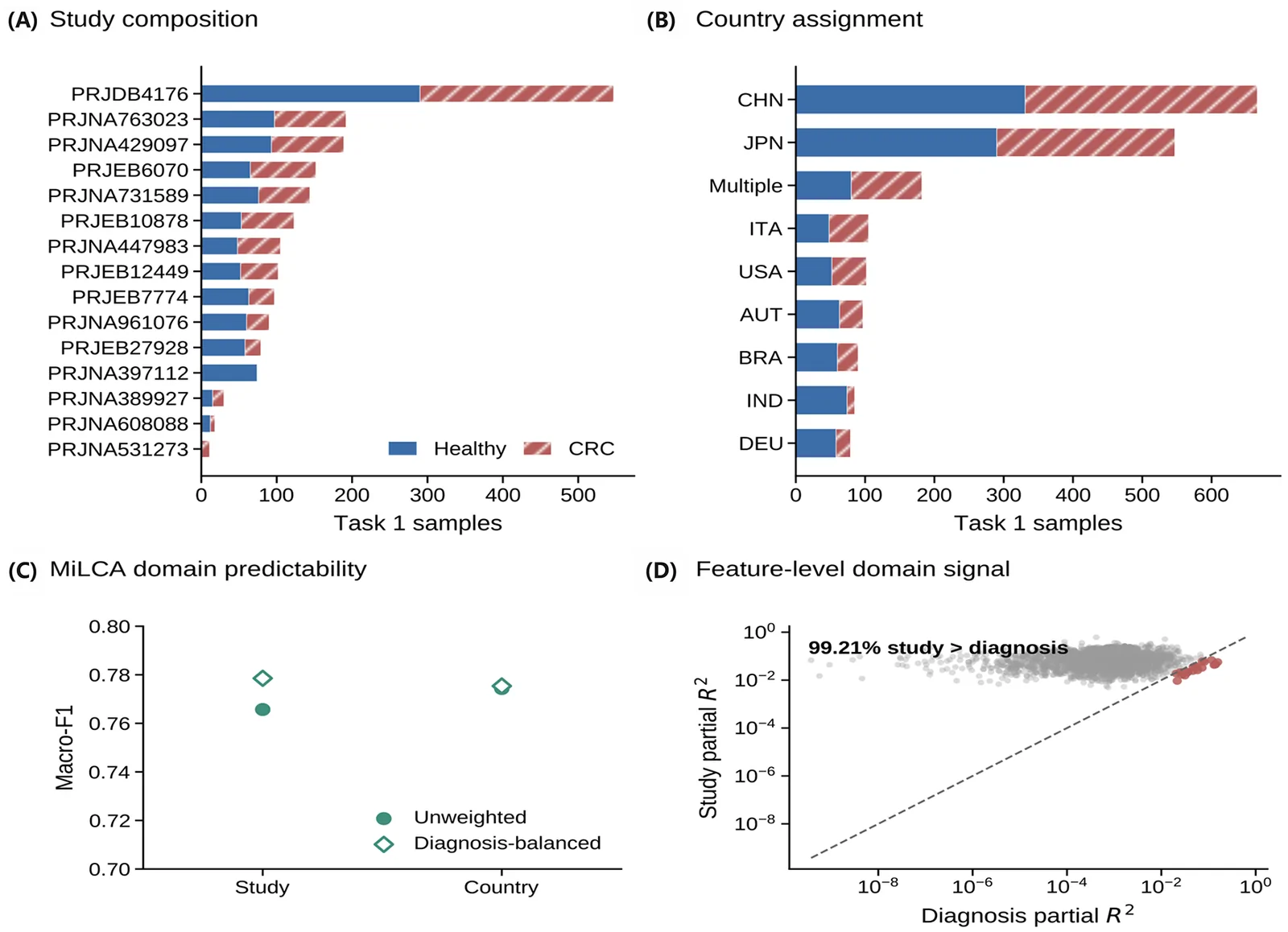
Cohort composition and domain structure in the Task 1 population (*n* = 1,953; 15 studies; 10 countries). **(A)** CRC and healthy samples across the 15 included studies. **(B)** Sample composition by country; “Multiple” denotes studies spanning more than one country. **(C)** MiLCA macro-F1 for study and country prediction before and after diagnosis balancing; higher macro-F1 indicates better domain classification. **(D)** Diagnosis-associated vs. study-associated partial *R*^2^ for the 2,543 taxonomic features. Points above the diagonal have larger study-associated effects. These partial effects describe domain structure; the comparison is descriptive, associational, and not complexity-adjusted.

### 4.2 RANDOM point estimates were higher than held-out-domain summaries

The single pre-specified subject-aware RANDOM split produced higher point estimates than held-out-domain evaluation (Table 2; Figure 3). M0 Elastic Net and M1 LightGBM achieved RANDOM AUROCs of 0.865 and 0.874. M0 macro AUROCs were 0.809 under LOSO and 0.782 under LOCO. For M1, the LOSO macro AUROC was 0.827 (exploratory between-study bootstrap interval, 0.782–0.874), and the LOCO macro AUROC was 0.821 (exploratory between-country bootstrap interval, 0.773–0.877). M2 cohort-balanced Elastic Net achieved a LOCO macro AUROC of 0.811. For M1, RANDOM and LOSO AUPRC values were 0.872 and 0.834, while the Brier score increased from 0.145 to 0.197. The pre-specified RANDOM split was retained as the primary in-distribution reference. Across 30 repeated subject-aware RANDOM splits, mean AUROC was 0.842 for M0 (SD 0.031; empirical P2.5–P97.5, 0.787–0.888) and 0.857 for M1 (SD 0.027; 0.808–0.904), compared with the respective single-split estimates of 0.865 and 0.874 (Supplementary Table S9). These sensitivity distributions quantify split-to-split variability and were not used to test RANDOM against LOSO or LOCO. Performance depended materially on the evaluation protocol, but none of these internal protocols constitutes independent prospective validation.

**Table 2:** Taxonomic predictive performance under the single pre-specified subject-aware RANDOM in-distribution split, leave-one-study-out (LOSO), and leave-one-country-out (LOCO) evaluation.

Protocol	Model	Evaluable domains	AUROC	AUPRC	Brier score	Worst AUROC	Worst domain
RANDOM	M0: Elastic Net	—	0.865	0.854	0.144	—	—
RANDOM	M1: LightGBM	—	0.874	0.872	0.145	—	—
LOSO	M0: Elastic Net	13/15	0.809	0.812	0.194	0.644	PRJNA389927
LOSO	M1: LightGBM	13/15	0.827 (0.782–0.874)	0.834	0.197	0.693	PRJNA389927
LOSO	M2: Cohort-balanced Elastic Net	13/15	0.816	0.818	0.194	0.644	PRJNA389927
LOCO	M0: Elastic Net	10/10	0.782	0.774	0.198	0.627	USA
LOCO	M1: LightGBM	10/10	0.821 (0.773–0.877)	0.803	0.180	0.697	JPN
LOCO	M2: Cohort-balanced Elastic Net	10/10	0.811	0.798	0.190	0.648	JPN

The RANDOM test role contained 205 samples (93 CRC and 112 healthy). LOSO and LOCO values are macro means across evaluable held-out domains. Parentheses for M1 AUROC give exploratory 95% between-domain percentile bootstrap intervals from 10,000 resamples of held-out studies (LOSO) or countries (LOCO); see Supplementary Table S14. Higher AUROC and AUPRC and lower Brier score indicate better performance. Two LOSO studies contained only one outcome class and therefore had AUROC and AUPRC recorded as not estimable.

**Figure 3:**
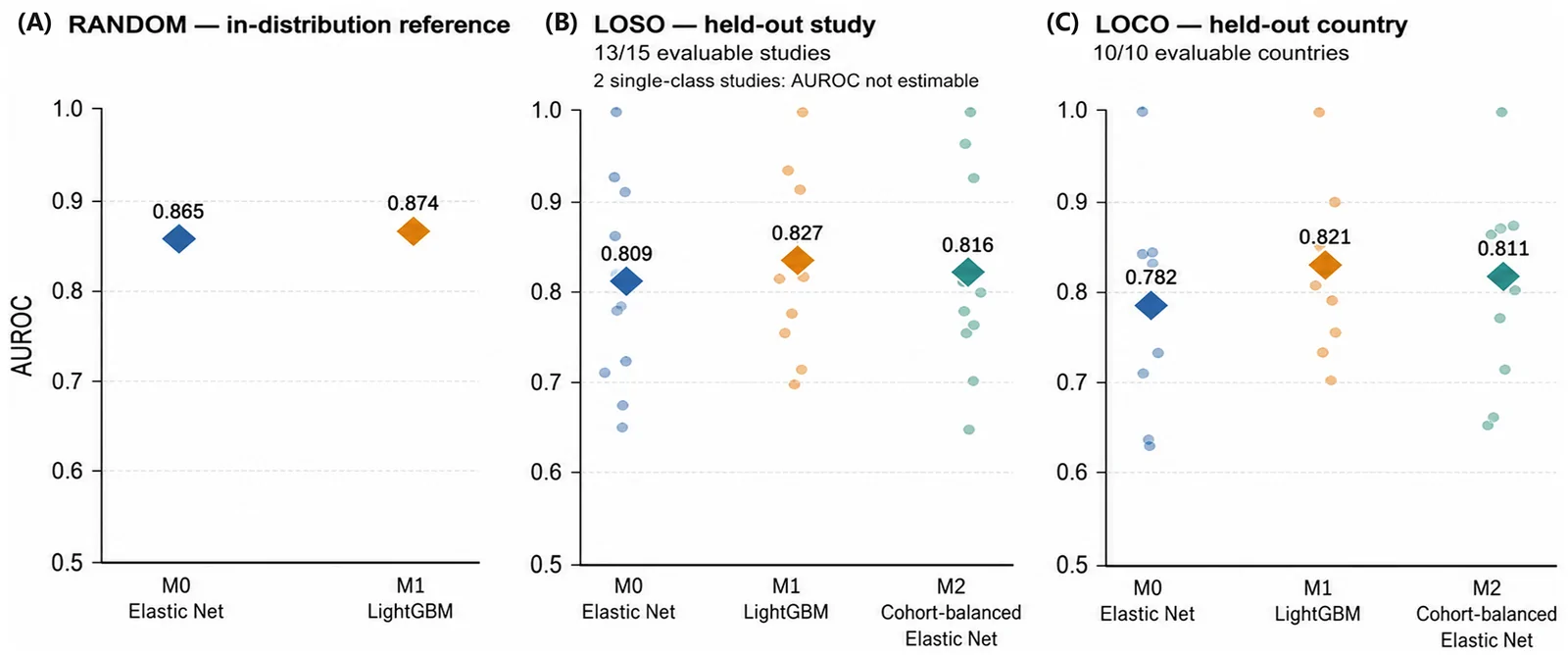
Taxonomic discrimination under RANDOM, leave-one-study-out (LOSO), and leave-one-country-out (LOCO) evaluation. **(A)** AUROC for the single pre-specified RANDOM split. Its test role contained 205 samples (93 CRC and 112 healthy). **(B)** LOSO AUROC across the 13 evaluable held-out studies; two single-class studies had AUROC recorded as not estimable. **(C)** LOCO AUROC across the ten held-out countries. Faint points denote held-out-domain AUROCs and diamonds denote the corresponding macro means. RANDOM, LOSO, and LOCO are internal evaluation protocols and do not constitute independent external validation. Higher AUROC indicates better discrimination. Repeated RANDOM sensitivity is reported in Supplementary Table S9 and is not shown here.

Two of the 15 held-out studies contained only one outcome class: PRJNA397112 included zero CRC and 74 healthy samples, whereas PRJNA531273 included 11 CRC and 0 healthy samples. Both AUROC and AUPRC were recorded as not estimable for these folds, leaving 13 evaluable LOSO folds (Supplementary Table S11). All ten LOCO folds contained both classes; their total sizes (CRC/healthy counts) were AUT 97 (34/63), BRA 90 (30/60), CAN 3 (1/2), CHN 666 (335/331), DEU 119 (56/63), FRA 112 (52/60), IND 85 (11/74), ITA 105 (57/48), JPN 547 (257/290), and USA 129 (64/65). Thirteen studies were single-country, while PRJEB6070 spanned DEU (40 samples) and FRA (112) and PRJNA389927 spanned CAN (3) and USA (27) (Supplementary Table S10). Across ten held-out countries, worst-country AUROC was 0.627 for M0, 0.697 for M1, and 0.648 for M2. Supplementary Table S2 summarizes RANDOM, LOSO, and LOCO performance, and Supplementary Table S14 reports exploratory domain-bootstrap uncertainty; complete fold-level outputs are provided with the machine-readable Supplementary material.

The pre-specified primary LOCO summaries retained all ten eligible countries, including CAN (three samples: one CRC and two healthy). Because this fold was small, we additionally summarized the same frozen country-level metrics after excluding CAN as a descriptive sensitivity; no model was retrained, and the primary LOCO protocol was unchanged. Primary vs. excluding-CAN taxonomic macro AUROCs were 0.782 vs. 0.758 for M0, 0.821 vs. 0.801 for M1, and 0.811 vs. 0.790 for M2; the corresponding AUPRCs were 0.774 vs. 0.749, 0.803 vs. 0.781, and 0.798 vs. 0.775. The taxonomic AUROC and AUPRC model rankings were unchanged, as were the worst-performing countries and their AUROCs (M0: USA, 0.627; M1: JPN, 0.697; M2: JPN, 0.648). The M1 LOCO bootstrap interval in Table 2 applies only to the primary ten-country analysis. Complete with- and without-CAN point summaries for the LOCO estimates reported in the article are provided in Supplementary Table S17 and the machine-readable Supplementary material.

### 4.3 Cohort structure was pervasive, whereas domain-robust learning provided limited benefit

MiLCA found substantial study and country information in the microbiome profiles (Figure 2). Microbiome-only study prediction yielded macro-F1 values of 0.766 before diagnosis balancing and 0.779 afterward; the corresponding country values were 0.774 and 0.775. In the pre-specified descriptive, associational, non-complexity-adjusted nested-model comparison, 99.21% of the 2,543 taxonomic features had a larger study-associated than diagnosis-associated partial effect. The median partial effect was 0.0677 for study and 0.0008 for diagnosis. Because the study and diagnosis terms differed substantially in dimensionality and parameterization, this directional comparison describes domain structure rather than statistical significance, causal attribution, or dominance of one source of variation. Metadata-only, missingness-only, diagnosis-balanced, and composition-restricted analyses also showed strong domain structure without attributing individual associations to geography, recruitment, laboratory processing, or another cause. Supplementary Table S3 summarizes the MiLCA and feature-level domain-structure audits; complete machine-readable audit outputs are provided with the Supplementary material.

Domain-robust training provided little additional benefit under LOSO (Figure 4). Macro AUROC was 0.816 for M2, 0.806 for M3 ERM-MLP, 0.811 for M4 cohort-balanced ERM-MLP, 0.812 for M5 Deep CORAL, and 0.806 for M6 GroupDRO, compared with 0.809 for M0 and 0.827 for M1. M2 exceeded M3 in 11 of 13 evaluable studies, but this paired difference was not supported after multiplicity correction [BH-adjusted (q = 0.160)]. None of the neural domain-robust methods consistently outperformed LightGBM. Supplementary Table S4 reports the domain-robust summaries and principal paired comparisons; complete fold- and seed-level outputs are provided with the Supplementary material.

**Figure 4:**
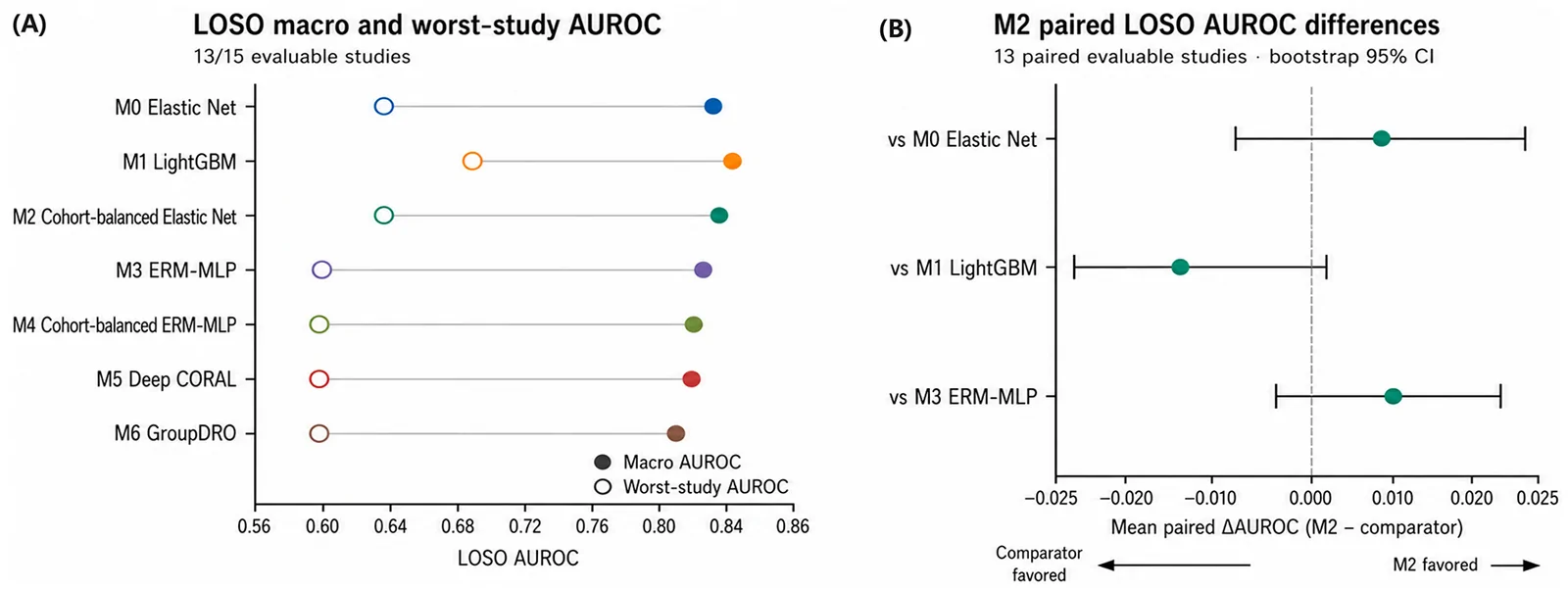
Baseline and domain-robust model comparisons under leave-one-study-out (LOSO) evaluation. Results are based on the 13 evaluable LOSO studies with both CRC and healthy samples. PRJNA397112 and PRJNA531273 were excluded from AUROC-based comparisons because their held-out folds contained only one outcome class. Higher AUROC indicates better discrimination. **(A)** Macro and worst-study AUROC for M0–M6. **(B)** Paired study-level AUROC differences between M2 and selected comparators with bootstrap 95% confidence intervals; positive values favor M2. LOSO is an internal held-out-study validation protocol within the retrospective MetaGenoPolis release and does not constitute independent external validation.

### 4.4 Calibration, conformal-style sets, and selective abstention characterized probability reliability

Domain-macro mean Brier scores and log losses decreased after Platt calibration for all three conventional models under LOCO evaluation (Table 3; Figure 5). Brier score decreased from 0.198 to 0.186 for M0, from 0.180 to 0.169 for M1, and from 0.190 to 0.175 for M2. Log loss decreased from 0.644 to 0.562, from 0.630 to 0.521, and from 0.609 to 0.534, respectively. Country-specific changes were heterogeneous, including worsening for some model–country pairs. Calibration parameters were estimated internally and were not refitted using held-out-country labels.

**Table 3:** Probability calibration, conformal-style coverage, and selective abstention under leave-one-country-out (LOCO) evaluation.

Model	Brier, raw	Brier, Platt	Log loss, raw	Log loss, Platt	Coverage, 90%	Set size, 90%	Coverage, 95%	Set size, 95%
A. Probability calibration and conformal-style sets								
M0	0.198	0.186	0.644	0.562	0.888	1.377	0.936	1.608
M1	0.180	0.169	0.630	0.521	0.869	1.267	0.931	1.512
M2	0.190	0.175	0.609	0.534	0.890	1.395	0.945	1.604
Model	Coverage, 80%	Error, 80%	Retention gap, 80%	Coverage, 90%	Error, 90%		Better than random, 80%	
B. Selective abstention and matched-random comparison						
M0	0.801	0.230	0.054	0.900	0.247		0.852	
M1	0.814	0.207	0.010	0.905	0.220		0.812	
M2	0.793	0.196	0.017	0.914	0.229		0.784	

M0 denotes Elastic Net, M1 LightGBM, and M2 cohort-balanced Elastic Net. Values are country-macro summaries across the ten LOCO folds. In panel **(A)**, coverage denotes empirical prediction-set coverage. In panel **(B)**, coverage denotes the retained sample proportion; retention gap is the absolute difference between CRC and healthy retention rates, and “better than random” is the fraction of 200 matched-random rejection repeats with higher error than uncertainty-based abstention. Lower Brier score, log loss, and selective error indicate better performance; conformal-style coverage is interpreted relative to its nominal target.

**Figure 5:**
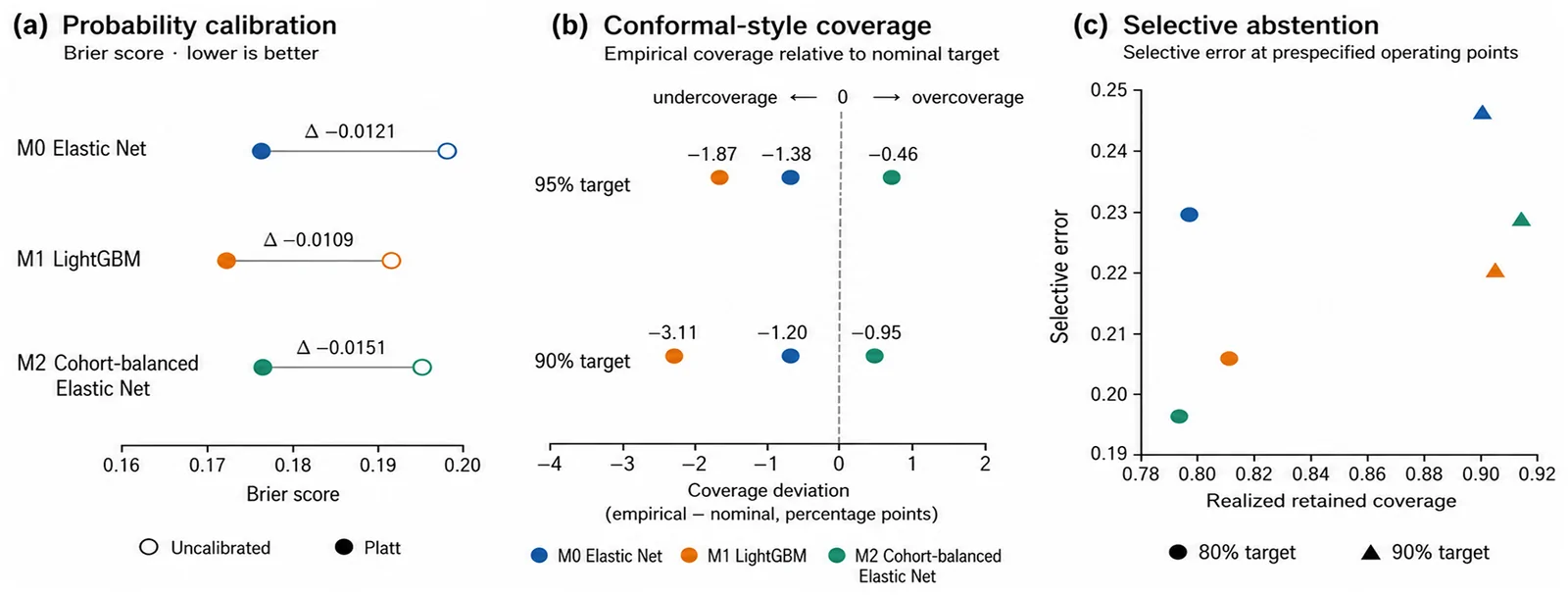
Probability reliability under leave-one-country-out (LOCO) evaluation. Values summarize the ten held-out countries using country-macro averages. **(a)** Domain-macro Brier scores before and after Platt calibration; lower values indicate better probability accuracy. **(b)** Deviation of empirical conformal-style coverage from the nominal 90 and 95% targets. **(c)** Selective error vs. realized retained coverage at the 80 and 90% target-coverage operating points; lower selective error is better. Calibration and uncertainty thresholds were estimated without using outer-test labels. Conformal-style coverage is reported empirically and does not constitute a finite-sample guarantee for future populations.

At nominal 90% conformal-style coverage, the domain-macro empirical value was 0.888 for M0, 0.869 for M1, and 0.890 for M2; at nominal 95%, coverage was 0.936, 0.931, and 0.945. These estimates describe empirical behavior under the recorded country-held-out protocol and are not guarantees for an unobserved future population; country-level results included material undercoverage. At the 90% selective-coverage target, selective error was 0.247, 0.220, and 0.229 for M0–M2; at the 80% target, it was 0.230, 0.207, and 0.196. For M1, uncertainty-based rejection produced lower error than matched-random rejection in 81.2% of repeats at the 80% target and in 75.4% of repeats at the 90% target. In the strict subject-disjoint Calibration-A/Calibration-B sensitivity, M1 primary vs. strict empirical coverage was 0.869 vs. 0.863 at nominal 90% and 0.931 vs. 0.937 at nominal 95%; Brier score was 0.169 vs. 0.171 and log loss was 0.521 vs. 0.525 (Supplementary Table S13). These results remained conformal-style empirical sensitivity summaries rather than a finite-sample guarantee for future domains. Supplementary Table S5 and Supplementary Figures S1, S2 summarize the primary reliability analyses, while Supplementary Tables S12–S14 report calibration sizes, strict results, and exploratory between-domain intervals; complete country-level outputs are provided with the Supplementary material.

### 4.5 Functional profiles were less predictive but more candidates met stability tiers

Functional profiles showed lower discrimination than the taxonomic representation across the pre-specified validation protocols. Functional RANDOM AUROC was 0.770 for M0, 0.751 for M1, and 0.766 for M2. Functional LOSO macro AUROC was 0.725, 0.739, and 0.720, whereas functional LOCO macro AUROC was 0.690, 0.743, and 0.626.

Figure 6 summarizes the corresponding cross-study stability analyses, including the numbers of taxonomic and functional candidates assigned to the pre-specified stability tiers and their median cross-study direction consistency. Numerically, more functional candidates met the pre-specified high-stability tiers: 1 Tier A and 46 Tier B functional candidates, compared with 7 Tier A and 33 Tier B taxonomic candidates. These raw counts are not directly comparable as discovery rates because the taxonomic and functional feature spaces differ in size and correlation structure. Predictive discrimination and cross-study stability therefore represented distinct properties.

**Figure 6:**
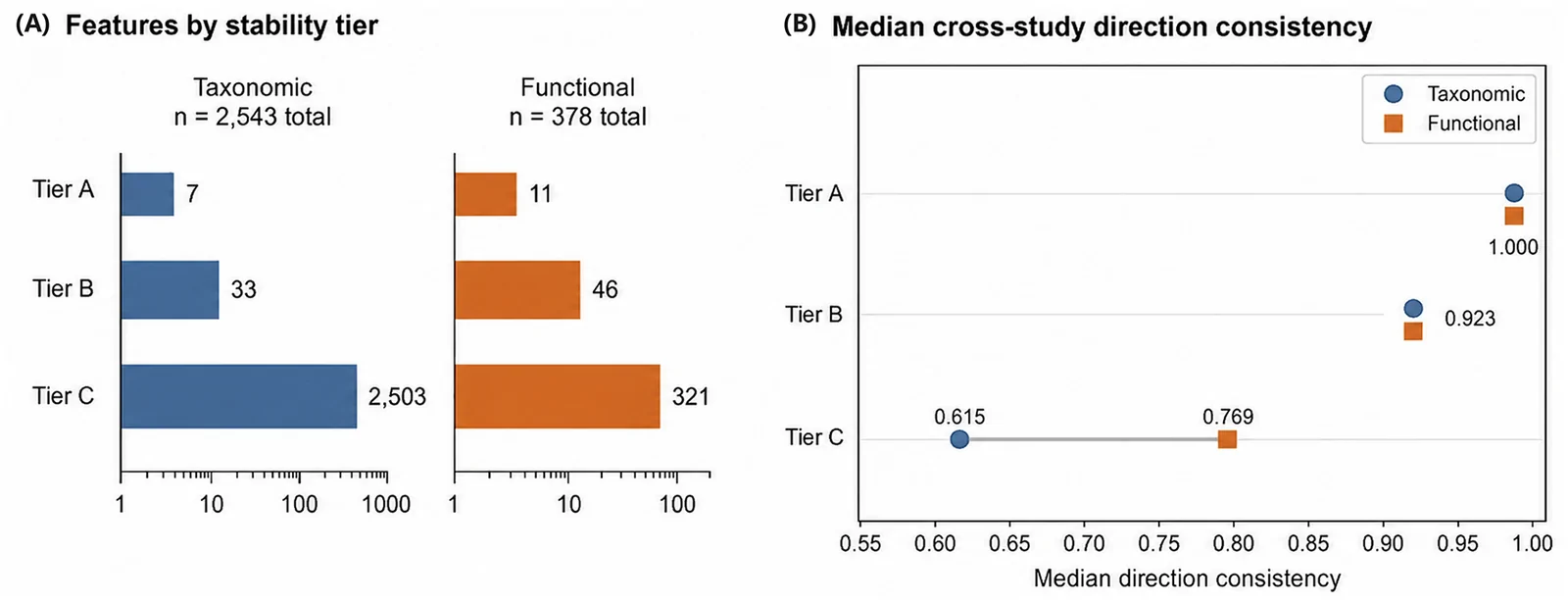
Cross-study stability of taxonomic and functional hypotheses. **(A)** Numbers of features assigned to pre-specified stability Tiers A–C. **(B)** Median cross-study direction consistency within each tier. Taxonomic and functional feature counts are not directly comparable as discovery rates because the feature spaces differ in size and correlation structure. The tiers identify stable candidate hypotheses rather than validated biomarkers.

Supplementary Table S6 summarizes functional discrimination and cross-study stability; complete taxonomic and functional candidate ledgers are provided with the Supplementary material. The retained features are stable candidate hypotheses rather than validated biomarkers or confirmed biological mechanisms; no independent cohort, raw-data reprocessing, or experimental validation was available.

### 4.6 The adenoma challenge and external-resource qualification defined the evidence boundary

Applied without refitting, the CRC-vs.-healthy models produced heterogeneous outputs for the 185 adenoma samples (Figure 7). Median calibrated CRC probabilities were 0.384 for M0, 0.356 for M1, and 0.350 for M2. Because adenoma was outside the binary outcome definition, we neither calculated ordinary binary accuracy nor optimized an adenoma threshold. Supplementary Table S7 summarizes the adenoma probability, prediction-set, and selective-retention results; complete adenoma predictions are provided with the Supplementary material.

**Figure 7:**
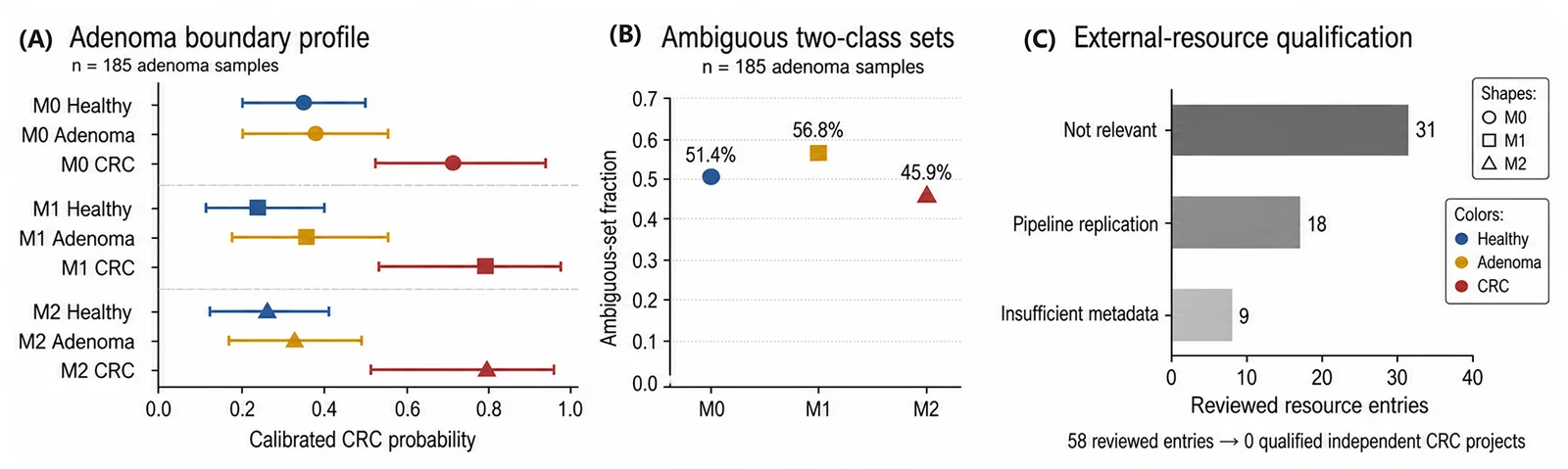
Adenoma challenge and external-resource qualification. **(A)** Median calibrated CRC probabilities and interquartile ranges for healthy, adenoma, and CRC samples under M0–M2. **(B)** Fraction of adenoma samples assigned ambiguous two-class prediction sets at nominal 90% coverage. **(C)** Qualification outcomes for the 58 reviewed external resources. The adenoma challenge comprised 185 samples and was outside the binary CRC-vs.-healthy outcome definition. No reviewed resource met all pre-specified requirements for formal independent CRC evaluation.

The external-resource assessment covered 58 project-level entries: 31 were not relevant to the primary task, 18 were pipeline replications, and nine lacked sufficient metadata. No resource satisfied the combined independence, licensing or authorization, and feature-compatibility criteria for formal external evaluation. The external allowlist was therefore empty, and no formal independent CRC or cross-disease prediction was performed. Supplementary Table S16 reports the complete resource-level audit and exclusion reasons. This is a limit of the recorded qualification exercise, not evidence of model failure on an eligible cohort or a universal absence of eligible resources.

## 5 Discussion

### 5.1 Generalization gaps, cohort shortcuts, and model robustness

The principal finding of CRC-TrustBench is that estimated performance depended materially on the evaluation protocol. For the primary LightGBM model, the single pre-specified RANDOM AUROC was 0.874, compared with 0.827 under LOSO evaluation and 0.821 under LOCO evaluation. These values should not be interpreted as competing estimates of one universal model performance. RANDOM splitting evaluated discrimination when training and test samples shared the study and country mixture represented in the source release, whereas LOSO and LOCO tested transfer to an unobserved study or country. The numerically higher RANDOM result provides an in-distribution reference, while the held-out-domain estimates provide more demanding assessments of transport within the retrospective collection. LOCO remains a country-label holdout within the same release, not independent external validation, and multi-country studies may retain study-associated processing characteristics across a country holdout. The exploratory LOCO bootstrap interval (0.773–0.877) included the pre-specified RANDOM point estimate of 0.874. This overlap does not establish statistical equivalence between the protocols: they target different generalization settings, and the interval summarizes variability across the observed held-out countries rather than uncertainty in a direct RANDOM–LOCO comparison.

Earlier metagenomic meta-analyses found reproducible CRC-associated microbial signals across cohorts (Wirbel et al., 2019; Thomas et al., 2019), and LightGBM retained meaningful discrimination under both LOSO and LOCO here. Across 30 repeated subject-aware RANDOM splits, AUROC varied around the single primary in-distribution reference (Supplementary Table S9). This sensitivity confirms split-to-split variability without making RANDOM, LOSO, and LOCO estimates statistically interchangeable; no cross-protocol significance test was performed.

MiLCA contextualized this sensitivity to the evaluation design. Microbiome profiles predicted study and country identity more accurately than diagnosis-only or metadata-only references, showing that potential shortcut information was available without proving that the CRC models used it. In the descriptive, associational, non-complexity-adjusted comparison, 99.21% of taxonomic features had a larger study-associated than diagnosis-associated partial effect. Because the terms differ in dimensionality and parameterization, this percentage is not a causal ranking. Geography, recruitment, host characteristics, sample handling, sequencing, and bioinformatic processing were partly aligned across cohorts, so the retrospective release cannot attribute the observed structure to one cause or label an individual feature as contaminated.

This domain structure is consistent with documented effects of specimen handling, measurement processes, and host covariates (Costea et al., 2017; McLaren et al., 2019; Vujkovic-Cvijin et al., 2020). MiLCA is diagnostic rather than corrective: it supports subject grouping, fold-local pre-processing, domain-held-out validation, and cautious interpretation, but does not justify retrospective feature removal.

Greater model complexity did not reliably improve held-out-domain performance. Cohort-balanced Elastic Net had a modest numerical advantage over the standard ERM multilayer perceptron in several held-out studies, but none of the neural domain-robust approaches consistently exceeded LightGBM in the pre-specified paired and multiplicity-adjusted comparisons. The limited study count, unequal cohort sizes, optimization variability, and heterogeneity that cannot be represented by a single removable domain direction may all contribute, although this experiment cannot separate their effects.

The pattern resembles broader domain-generalization benchmarks, where complex invariance objectives often offer limited gains once model selection and strong empirical-risk-minimization baselines are controlled (Gulrajani and Lopez-Paz, 2021; Koh et al., 2021; Zhou et al., 2023). Only 13 studies provided evaluable binary AUROC in CRC-TrustBench, leaving high-capacity models relatively little domain-level information from which to learn stable invariances. Study balancing can reduce unequal contributions to the training objective, but it cannot make countries exchangeable, recover unmeasured covariates, or separate technical effects from biological variation. LightGBM remained the strongest model in the benchmark; the domain-robust models supplied bounded comparisons rather than a replacement for conventional baselines.

### 5.2 Probability reliability under cohort shift

Discrimination alone did not provide a complete description of model behavior under country-level shift. Domain-macro mean Brier scores and log losses decreased after Platt scaling for all three conventional models, with the primary LightGBM Brier score decreasing from 0.180 to 0.169 and log loss decreasing from 0.630 to 0.521. Country-specific changes were heterogeneous and included deterioration for some model–country pairs. These aggregate changes occurred without changing the underlying ranking experiment measured by AUROC. A model can therefore retain similar discrimination while producing probabilities that differ materially in their agreement with observed outcome frequencies.

For LightGBM, domain-macro empirical coverage at the nominal 90% conformal-style level was 0.869 rather than exactly 0.900, and some countries showed material undercoverage. Standard split-conformal guarantees depend on a predictor fixed independently of the score-calibration labels and on exchangeability between calibration and future observations (Vovk et al., 2005; Angelopoulos and Bates, 2023). Here, the calibration records were reused for Platt fitting and score quantiles, and a held-out country can also differ systematically in population composition, recruitment, and processing. The reported values are therefore descriptive coverage under the recorded LOCO protocol, not finite-sample guarantees for arbitrary future countries or assay pipelines. A strict subject-disjoint calibration sensitivity produced materially similar empirical conclusions while retaining country-level heterogeneity; it does not establish a future-domain guarantee.

Selective abstention showed that errors were concentrated among uncertain predictions. At the 80% target coverage, the LightGBM accepted subset had a selective error of 0.207, and uncertainty-based rejection produced lower error than matched-random rejection in most recorded repeats. Matching the number of accepted samples separated the value of the uncertainty score from the mechanical effect of removing observations, a pattern consistent with the score capturing prediction difficulty within the benchmark.

These operating characteristics do not define a clinical referral or diagnostic workflow: rejected samples require an explicit downstream action, and class-specific retention requires monitoring. Because predictive uncertainty may deteriorate under dataset shift (Ovadia et al., 2019), calibration, empirical prediction sets, and abstention characterize this benchmark rather than establish screening readiness or clinical utility.

### 5.3 Stable candidate hypotheses and the adenoma challenge

Taxonomic and functional analyses distinguished sample-level discrimination from cross-cohort stability. Functional profiles had lower predictive AUROC than taxonomic profiles, but more functional candidates met Tier A and Tier B: 11 and 46, compared with seven and 33 taxonomic candidates. These raw counts do not establish a higher discovery rate because the feature spaces differ in size and correlation structure. A representation may therefore classify individual samples less effectively while still yielding candidates that satisfy pre-specified cross-study criteria.

Functional aggregation may reduce species-level variation and partly buffer population differences in taxonomic composition. Yet functional profiles remain sensitive to database coverage, annotation quality, correlated pathways, and the resolution of the source representation (Beghini et al., 2021; Franzosa et al., 2019). The larger number of stable functional candidates neither confirms their mechanisms nor establishes that functional profiles are intrinsically superior.

The tiering procedure combined meta-analytic evidence, direction consistency, heterogeneity, leave-one-study-out influence, and bootstrap stability, reducing the chance that a candidate reflected one large cohort or an unstable model fit. It cannot establish biochemical activity, disease specificity, or causality. The retained features are stable candidate hypotheses for independent measurement, targeted replication, or experimental perturbation, not validated biomarkers; no independent cohort, raw-data reprocessing, or experimental validation was available.

The adenoma analysis addressed a different boundary. Adenoma was neither a healthy-control nor a CRC label in the binary training task, and the 185 eligible adenoma samples were excluded from fitting, selection, calibration, and uncertainty-threshold estimation. The primary LightGBM model assigned a median calibrated CRC probability of 0.356, with substantial variation across samples. This distribution describes how the fixed CRC-vs.-healthy classifier responded to an intermediate challenge population; it does not define adenoma classification accuracy or a disease-progression score.

Previous studies have reported microbiome changes across the adenoma–carcinoma sequence (Feng et al., 2015; Yachida et al., 2019), but a probability produced by a binary CRC-vs.-healthy model is not a calibrated measure of progression. Lesion stage, histopathological subtype, longitudinal outcome, and an adenoma-specific target were not available in a form sufficient to define such an estimand. The adenoma results should therefore not be converted into screening sensitivity, false-positive rates, patient risk, or a threshold for clinical action. Their value is to expose model behavior outside the binary training definition while preserving the distinction between challenge analysis and validation.

### 5.4 Strengths, limitations, and future validation priorities

Several design choices strengthen CRC-TrustBench. Population eligibility, label harmonization, subject grouping, feature-matrix governance, and split assignments were fixed before the final comparisons. Globally corrected or filtered matrices that could transfer information across folds were blocked, and all sample-dependent pre-processing was fitted within the corresponding training partition. RANDOM, LOSO, and LOCO were reported as distinct estimands, calibration data were separated from validation and outer-test data, and single-class domains remained in the analysis record with non-estimable metrics identified. Negative and non-evaluable findings, including the limited gains of domain-robust methods and the absence of an eligible external evaluation resource, were retained.

The study nevertheless remains a retrospective secondary analysis of one curated public release, with unequal study sizes, incomplete clinical and technical metadata, and a limited number of independent studies and countries. Two LOSO folds contained only one outcome class and could not provide binary AUROC. Important covariates, including age, sex, medication, disease stage, diet, recruitment setting, and sequencing protocol, were not uniformly available. Because released processed profiles rather than uniformly reprocessed raw FASTQ data were analyzed, observed study effects may reflect both biological and technical variation. The functional analysis was also restricted to the available 378-feature representation. Stable candidate hypotheses lacked orthogonal experimental validation, MiLCA identified domain structure without resolving its cause, and the empirical prediction-set and abstention results apply only to the recorded calibration and LOCO design.

External-resource qualification defined an additional evidence boundary. No reviewed resource simultaneously satisfied sample-level independence, authorized use, and compatibility with the frozen feature space; pipeline replications were not treated as independent cohorts. Public availability alone does not establish independence, phenotype quality, permitted use, or feature compatibility (Pasolli et al., 2017; Liu et al., 2026). Consequently, no qualified independent test was performed, which is not evidence of model failure. The models were not directly compared with established CRC screening approaches; discrimination, calibration, and abstention do not establish clinical utility, and CRC-TrustBench is a methodological benchmark rather than a clinical deployment study (Collins et al., 2015; Wolff et al., 2019).

Future work should prioritize independent validation before expanding the model search. Suitable external cohorts should provide auditable sample identities, appropriate authorization, compatible or reproducibly mappable features, and sufficiently detailed phenotypes. Frozen pre-processing and models should then be applied without external tuning, recalibration, or feature reselection unless pre-specified as a separate adaptation study. Prospective and cross-disease evaluations are also needed to assess transportability, disease specificity, and the handling of uncertain predictions, while stable candidate hypotheses require independent replication and experimental assessment. Recent reanalysis and retraction of cancer microbiome findings illustrate how analytical and normalization errors can alter apparently strong results (Gihawi et al., 2023; Poore et al., 2024). Subject grouping, namespaced identifiers, fold-local pre-processing, immutable splits, and explicit evidence gates reduce these risks but cannot compensate for incomplete source records. CRC-TrustBench separates in-distribution discrimination, cross-domain generalization, probability reliability, hypothesis stability, and external-resource eligibility.

## 6 Conclusion

CRC-TrustBench establishes an international public CRC metagenomic benchmark with explicit adjudication, leakage-safe splits, domain analyses, calibrated uncertainty, and traceable stability analyses. The single pre-specified RANDOM estimate was numerically higher than the held-out-study and held-out-country summaries; LightGBM remained the primary discriminative reference; calibration and selective abstention added information about probability reliability; more functional candidates met the pre-specified stability tiers despite lower predictive performance; and adenoma outputs were heterogeneous. No reviewed external resource qualified under the recorded criteria. The benchmark supports auditable model assessment and hypothesis generation, but not clinical deployment, causal inference, or claims of independent external generalization.

## Data Availability

Publicly available datasets were analyzed in this study. This data can be found here: MetaGenoPolis V16.0 data release (Barbet et al., 2022): https://doi.org/10.57745/7IVO3E. Repository: MetaGenoPolis. DOI: 10.57745/7IVO3E. Derived reproducibility materials are available in the CRC-TrustBench Zenodo repository, version V1.0.1: https://doi.org/10.5281/zenodo.22346874.
