# Correction: CRC-TrustBench: benchmarking cross-study and cross-country generalization in gut metagenomic colorectal cancer prediction

**DOI:** 10.3389/fmicb.2026.2021752

**Published:** 2026-10-16

**Authors:** Yuzheng Zhang, Yangzong Cidan, Jiayi Xu, Bingtong Li

**Affiliations:** 1 Department of Hospital Infection Management, China-Japan Union Hospital of Jilin University, Changchun, China; 2 Department of Epidemiology and Biostatistics, School of Public Health, Jilin University, Changchun, China; 3 Department of Scientific Research, China-Japan Union Hospital of Jilin University, Changchun, China; 4 Department of Rheumatology and Immunology, China-Japan Union Hospital of Jilin University, Changchun, China

**Keywords:** colorectal cancer, cross-cohort generalization, data leakage, gut metagenomics, uncertainty-aware prediction

An incorrect number was provided for [242730XM0103118292]. The correct number is [YDZJ202501ZYTS100].

The original version of this article has been updated.

