# Supplementary material for: CRC-TrustBench: benchmarking cross-study and cross-country generalization in gut metagenomic colorectal cancer prediction
Source: Front Microbiol. 2026 Sep 25;17:1971707. doi: 10.3389/fmicb.2026.1971707 (PMC13650428; doi:10.3389/fmicb.2026.1971707)
Supplement: Supplementary file 1 [file Data_Sheet_1.pdf]

Supplementary Material

1 Supplementary Methods

1.1 Source data, population governance, and leakage control

The analysis used the MetaGenoPolis V16.0 processed-profile release. Source identifiers and retrieval information were registered before analysis. Diagnostic fields were retained before mapping to colorectal cancer (CRC), healthy, adenoma, other disease, or unknown. Repeated observations were reconciled at the subject-group level before split construction. For PRJDB4176, the presurgical *t1* record was used in the primary analysis and postsurgical *t2* observations were reserved for sensitivity interpretation. Only the uncorrected 2,543-feature taxonomic matrix and the 378-feature functional matrix were permitted for modeling; 28 globally corrected or filtered matrices remained blocked. Prevalence filtering, zero replacement, centered log-ratio transformation, scaling, and model-specific feature selection were fitted within the corresponding training partition.

1.2 Validation protocols and predictive models

RANDOM used immutable subject-aware training, validation, calibration, and test roles and served as an in-distribution reference. Leave-one-study-out (LOSO) held out each of 15 studies; two folds contained only one outcome class, so the area under the receiver operating characteristic curve (AUROC) and area under the precision-recall curve (AUPRC) were not estimable for those folds. Leave-one-country-out (LOCO) held out each of 10 countries. M0 Elastic Net, M1 LightGBM, and M2 cohort-balanced Elastic Net formed the conventional comparison. M3 empirical-risk-minimization multilayer perceptron (ERM-MLP), M4 cohort-balanced ERM-MLP, M5 Deep CORAL, and M6 GroupDRO were prespecified domain-robust comparisons. Outer-test data were unavailable to preprocessing, model selection, calibration, and threshold estimation.

1.3 MiLCA and domain-structure audits

The Microbiome Leakage and Confounding Audit (MiLCA) evaluated whether study or country identity could be predicted from microbiome profiles and from prespecified metadata-based views. Diagnosis-balanced analyses tested whether domain predictability could be explained solely by diagnosis imbalance. Feature-level study-associated and diagnosis-associated partial effects were descriptive audit quantities and were not used for retrospective feature removal.

1.4 Probability calibration and uncertainty analyses

Within LOCO, Platt scaling was fitted using the reserved calibration role. Conformal-style sets used calibrated probabilities and the prespecified finite-sample score-quantile rule at nominal 90% and 95% coverage. Because the primary analysis reused the calibration records for Platt fitting and score quantiles, and because held-out countries may violate exchangeability, reported coverage was interpreted empirically rather than as a future-domain finite-sample guarantee. A strict subject-disjoint Calibration-A/Calibration-B sensitivity was analyzed separately. Selective abstention used predictive entropy with calibration-derived target coverages; matched-random rejection used 200 fixed-seed repeats with the same accepted sample count.

1.5 Stable hypotheses and boundary analyses

Taxonomic and functional candidates were tiered using within-study effects, random-effects synthesis, direction consistency, heterogeneity, leave-one-study-out influence, and subject-group bootstrap stability. Tier labels denote cross-cohort hypothesis stability rather than validated biomarkers or mechanisms. The 185 quality-controlled adenoma samples were evaluated only after Task 1 models were frozen and were not assigned binary correctness. External resources were screened using a conjunctive gate requiring sample-level independence, authorization, and compatibility with the frozen feature space.

1.6 Statistical summaries and reproducibility

Domain-macro summaries used held-out studies or countries as the aggregation unit. Paired comparisons matched methods within the same held-out domain and retained prespecified multiplicity adjustment. Metrics that were not estimable remained distinct from zero. Machine-readable data supporting the additional sensitivity analyses and audit summaries are provided in the Supplementary Data directory.

2 Supplementary Results

2.1 Population and split integrity

Task 1 contained 1,953 subject-grouped samples (897 CRC and 1,056 healthy) across 15 studies and 10 countries. Addition of 185 adenoma samples yielded the 2,138-sample quality-controlled Task 2 population. Study-level composition and quality-control exclusions are summarized in Supplementary Table S1. No subject group crossed a fixed analytical partition.

2.2 Held-out-domain performance

The single prespecified RANDOM point estimates were higher than the corresponding held-out-domain summaries for the principal conventional models. LOSO retained 13 evaluable study-level AUROCs, and all 10 country folds were evaluable for LOCO. Supplementary Table S2 reports macro, dispersion, and worst-domain summaries, and Supplementary Table S11 reports class composition and metric evaluability. The CAN fold contained only three samples (one CRC and two healthy). The prespecified primary LOCO summaries retained all 10 countries; a descriptive re-aggregation excluding CAN, based on the same frozen country-level metrics and without model retraining, is reported in Supplementary Table S17 and the machine-readable Supplementary Data.

2.3 MiLCA and domain structure

Microbiome-only MiLCA macro-F1 was 0.766 for study and 0.774 for country under unweighted training, and 0.779 and 0.775 after diagnosis-balanced training. In the descriptive, non-complexity-adjusted feature-level audit, study-associated partial effects exceeded diagnosis-associated partial effects for 99.21% of the 2,543 taxonomic features. Supplementary Table S3 summarizes these audit endpoints. These results establish the availability of domain information but do not identify its biological or technical cause and do not prove that CRC classifiers used the same information.

2.4 Domain-robust comparisons

Across 13 evaluable LOSO studies, M1 LightGBM had the highest macro AUROC among M0–M6. The paired M2 comparisons did not establish multiplicity-adjusted superiority over the selected comparators. Supplementary Table S4 reports model summaries and the principal paired contrasts.

2.5 Calibration, conformal-style sets, and selective abstention

Platt scaling reduced domain-macro mean Brier score and log loss for all three conventional models. Empirical conformal-style coverage remained below nominal levels for several model–target combinations, and the strict disjoint-calibration sensitivity produced similar qualitative conclusions. Uncertainty-based abstention produced lower error than matched-random rejection in most recorded repeats at the prespecified 80% and 90% target coverages. Supplementary Table S5 summarizes the numerical results, Supplementary Figure S1 shows LightGBM calibration behavior, and Supplementary Figure S2 shows the strict conformal sensitivity.

2.6 Functional benchmarking and stable hypotheses

Functional profiles showed lower discrimination than the taxonomic representation across the prespecified protocols. More functional candidates met Tier A and Tier B, but raw counts are not directly comparable as discovery rates because the feature spaces differ in size and correlation structure. Supplementary Table S6 reports functional AUROC summaries and stability-tier statistics.

2.7 Adenoma and external-resource boundaries

Adenoma CRC probabilities were heterogeneous across the frozen models, and no binary adenoma accuracy was defined. Among 58 reviewed external-resource entries, 31 were not relevant to the primary task, 18 were pipeline replications, and nine had insufficient metadata; none qualified for formal independent evaluation. Supplementary Table S7 summarizes both boundary analyses.

3 Supplementary Figures

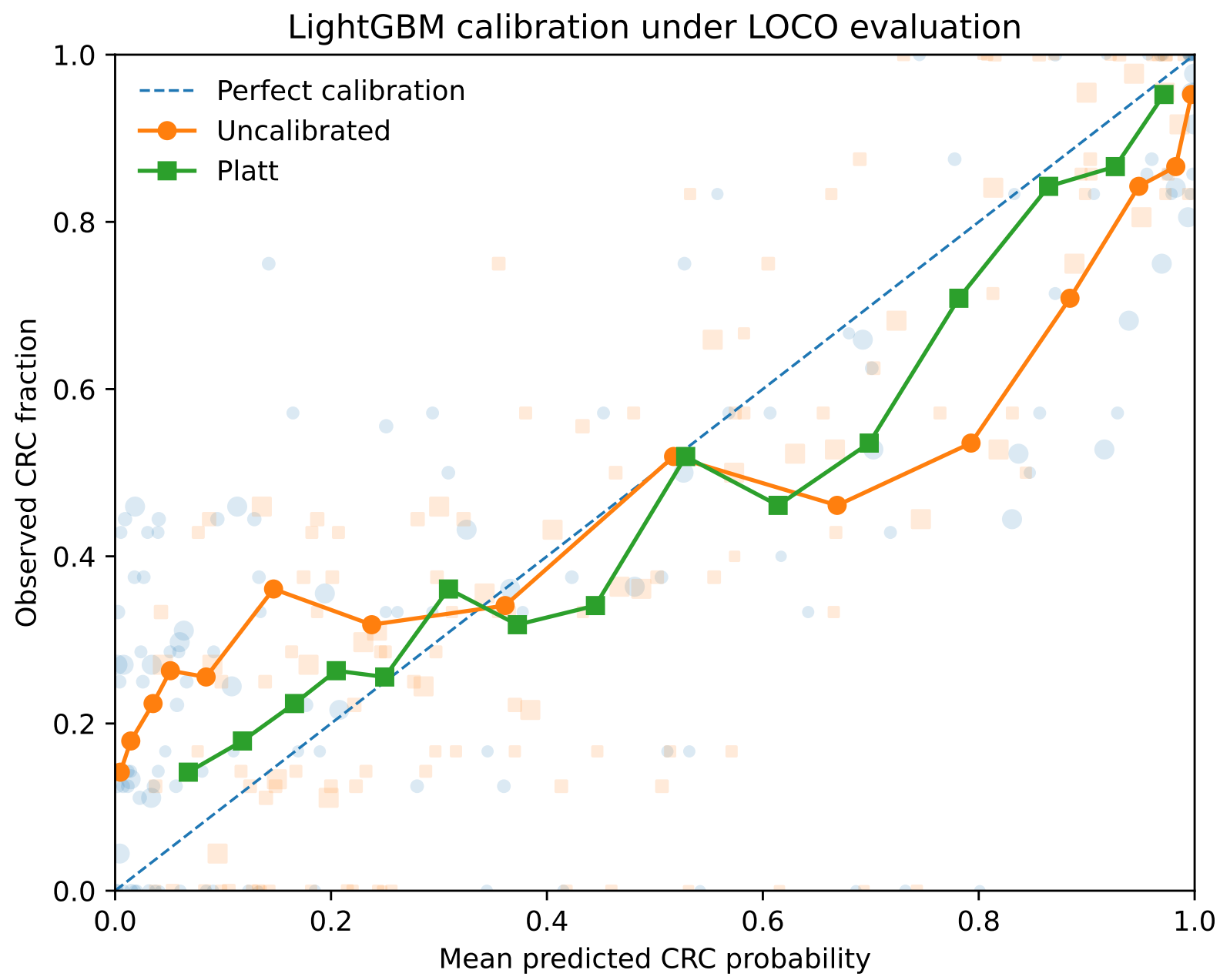

Figure S1: LightGBM calibration under leave-one-country-out evaluation. Faint points show country-level reliability bins before and after Platt scaling; marker area reflects bin size. Lines connect bin-number summaries weighted by the number of observations across countries. The diagonal denotes perfect calibration. The figure is descriptive of the recorded LOCO protocol and is not an external calibration guarantee.

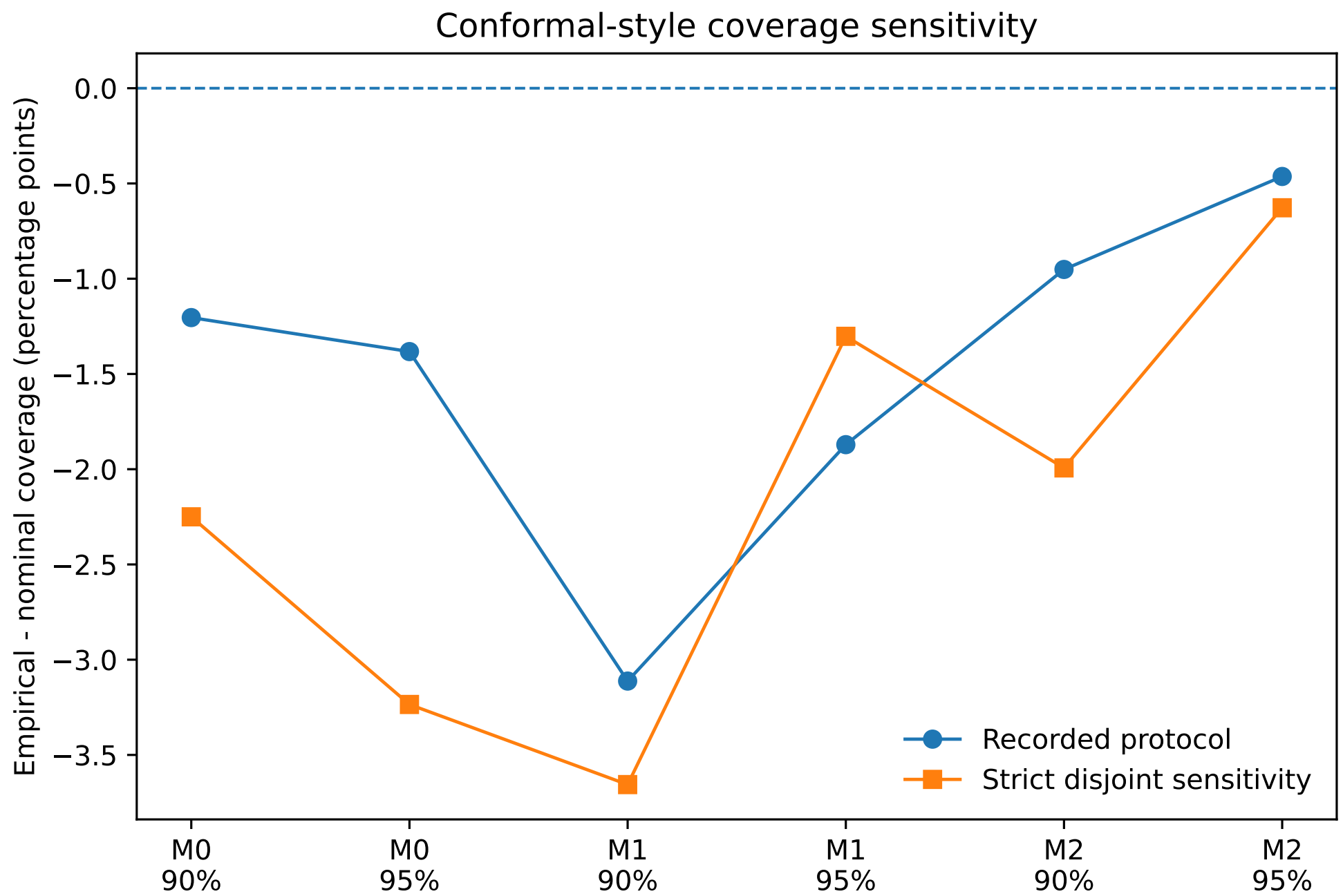

Figure S2: Strict disjoint-calibration sensitivity for conformal-style coverage. Values are domain-macro empirical coverage minus nominal coverage for the recorded trust-layer analysis and the subject-disjoint Calibration-A/Calibration-B sensitivity. Negative values denote undercoverage. The sensitivity analysis did not establish a future-domain coverage guarantee.

4 Supplementary Tables

Table S1: Study-level composition and quality-control summary. Task 1 contains CRC and healthy samples only; adenoma samples contribute only to the quality-controlled Task 2 challenge population. QC exclusions are recorded source-study exclusions under the frozen adjudication policy.

Study	Country	Task 1, n	CRC	Healthy	Adenoma	QC eligible, n	QC exclusions
PRJDB4176	JPN	547	257	290	67	614	31
PRJEB10878	CHN	123	70	53	0	123	5
PRJEB12449	USA	102	50	52	0	102	2
PRJEB27928	DEU	79	21	58	0	79	3
PRJEB6070	MULTIPLE	152	87	65	37	189	10
PRJEB7774	AUT	97	34	63	46	143	13
PRJNA389927	MULTIPLE	30	15	15	9	39	45
PRJNA397112	IND	74	0	74	0	74	36
PRJNA429097	CHN	189	96	93	0	189	4
PRJNA447983	ITA	105	57	48	26	131	9
PRJNA531273	IND	11	11	0	0	11	19
PRJNA608088	CHN	18	6	12	0	18	0
PRJNA731589	CHN	144	68	76	0	144	17
PRJNA763023	CHN	192	95	97	0	192	8
PRJNA961076	BRA	90	30	60	0	90	0
Total	-	1,953	897	1,056	185	2,138	202

Table S2: Taxonomic predictive performance under the prespecified validation protocols. LOSO and LOCO values are arithmetic domain-macro summaries. RANDOM is the single prespecified in-distribution split. SD and worst-domain AUROC are not defined for the single RANDOM split.

Protocol	Model	Evaluable domains	AUROC	AUPRC	Brier	Median AUROC	AUROC SD	Worst AUROC
RANDOM	M0 Elastic Net	single split	0.865	0.854	0.144	–	–	–
RANDOM	M1 LightGBM	single split	0.874	0.872	0.145	–	–	–
LOSO	M0 Elastic Net	13/15	0.809	0.812	0.194	0.819	0.104	0.644
LOSO	M1 LightGBM	13/15	0.827	0.834	0.197	0.819	0.087	0.693
LOSO	M2 cohort-balanced Elastic Net	13/15	0.816	0.818	0.194	0.809	0.100	0.644
LOCO	M0 Elastic Net	10/10	0.782	0.774	0.198	0.812	0.107	0.627
LOCO	M1 LightGBM	10/10	0.821	0.803	0.180	0.824	0.084	0.697
LOCO	M2 cohort-balanced Elastic Net	10/10	0.811	0.798	0.190	0.847	0.105	0.648

Table S3: MiLCA domain-predictability and feature-level domain-structure summaries. Panel A reports microbiome-only identity prediction using the raw evaluation weighting. Panel B reports descriptive feature-level partial-effect summaries.

A. Microbiome-only domain predictability

Target	Training	Macro-F1	Balanced accuracy	Top-1 accuracy
Study	Unweighted	0.766	0.760	0.878
Study	Diagnosis-balanced	0.779	0.777	0.879
Country	Unweighted	0.774	0.769	0.921
Country	Diagnosis-balanced	0.775	0.776	0.921

B. Feature-level domain structure

Metric	Value
Taxonomic features with finite partial effects	2,543
Study-associated effect > diagnosis-associated effect	99.21%
Median study-associated partial R^2	0.0677
Median diagnosis-associated partial R^2	0.0008

Table S4: LOSO domain-robust model summaries and principal paired M2 contrasts. The paired differences are study-level AUROC differences; positive values favor M2. Improved/worsened/tied counts refer to the 13 evaluable study folds.

A. Model summaries

Model	Macro AUROC	Median	SD	Worst AUROC	Worst study
M0 Elastic Net	0.809	0.819	0.104	0.644	PRJNA389927
M1 LightGBM	0.827	0.819	0.087	0.693	PRJNA389927
M2 cohort-balanced Elastic Net	0.816	0.809	0.100	0.644	PRJNA389927
M3 ERM-MLP	0.806	0.800	0.105	0.610	PRJNA389927
M4 cohort-balanced ERM-MLP	0.811	0.806	0.103	0.610	PRJNA389927
M5 Deep CORAL	0.812	0.806	0.103	0.610	PRJNA389927
M6 GroupDRO	0.806	0.800	0.105	0.612	PRJNA389927

B. Paired contrasts

Contrast	Mean Δ AUROC	Bootstrap 95% CI	Sign-flip p	Wilcoxon q (BH-adjusted)	I/W/T
M2 - M0	+0.008	[-0.008, +0.024]	0.399	0.677	7/5/1
M2 - M1	-0.011	[-0.023, +0.001]	0.121	0.420	5/7/1
M2 - M3	+0.011	[-0.003, +0.021]	0.118	0.160	11/1/1

Table S5: Country-held-out calibration, conformal-style coverage, and selective abstention. Values are arithmetic domain-macro summaries across the ten LOCO folds. Better-than-random is the mean fraction of 200 matched-random rejection repeats with higher error than uncertainty-based abstention.

A. Probability calibration

Model	Brier raw	Brier Platt	Log loss raw	Log loss Platt
M0 Elastic Net	0.198	0.186	0.644	0.562
M1 LightGBM	0.180	0.169	0.630	0.521
M2 cohort-balanced Elastic Net	0.190	0.175	0.609	0.534

B. Conformal-style sets

Model	Nominal coverage	Empirical coverage	Mean set size	Singleton fraction
M0 Elastic Net	90%	0.888	1.377	0.623
M0 Elastic Net	95%	0.936	1.608	0.392
M1 LightGBM	90%	0.869	1.267	0.733
M1 LightGBM	95%	0.931	1.512	0.488
M2 cohort-balanced Elastic Net	90%	0.890	1.395	0.605
M2 cohort-balanced Elastic Net	95%	0.945	1.604	0.396

C. Selective abstention

Model	Target coverage	Realized coverage	Selective error	Better than random
M0 Elastic Net	80%	0.801	0.230	85.2%
M0 Elastic Net	90%	0.900	0.247	78.0%
M1 LightGBM	80%	0.814	0.207	81.2%
M1 LightGBM	90%	0.905	0.220	75.4%
M2 cohort-balanced Elastic Net	80%	0.793	0.196	78.5%
M2 cohort-balanced Elastic Net	90%	0.914	0.229	67.0%

Table S6: Functional discrimination and cross-study stability summaries. Functional AUROCs are the prespecified protocol summaries reported in the benchmark. Stability counts and medians are not directly comparable discovery rates across feature domains because the feature spaces differ in size and correlation structure.

A. Functional discrimination

Protocol	Model	AUROC
RANDOM	M0 Elastic Net	0.770
RANDOM	M1 LightGBM	0.751
RANDOM	M2 cohort-balanced Elastic Net	0.766
LOSO	M0 Elastic Net	0.725
LOSO	M1 LightGBM	0.739
LOSO	M2 cohort-balanced Elastic Net	0.720
LOCO	M0 Elastic Net	0.690
LOCO	M1 LightGBM	0.743
LOCO	M2 cohort-balanced Elastic Net	0.626

B. Stability tiers

Feature domain	Tier	Candidates, n	Direction consistency	Median I^2, %	Median BH q
Taxonomic	A	7	1.000	7.0	1.44e-09
Taxonomic	B	33	0.923	47.3	0.000203
Taxonomic	C	2,503	0.615	43.4	0.869
Functional	A	11	1.000	25.1	1.8e-06
Functional	B	46	0.923	41.6	0.000424
Functional	C	321	0.769	50.1	0.0635

Table S7: Adenoma challenge and external-resource qualification. Adenoma outputs are descriptive because adenoma was not a class in the binary CRC-versus-healthy task. External-resource qualification required independence, authorization, and feature compatibility simultaneously.

A. Adenoma challenge ($n = 185$)

Model	n	Median CRC probability	IQR	Ambiguous set, 90%	Accepted, 80% target	Accepted, 90% target
M0 Elastic Net	185	0.384	0.253–0.533	51.4%	68.6%	83.2%
M1 LightGBM	185	0.356	0.205–0.510	56.8%	67.6%	85.4%
M2 cohort-balanced Elastic Net	185	0.350	0.203–0.480	45.9%	71.4%	85.9%

B. External-resource qualification

Outcome	Project-level entries, n
Not relevant	31
Pipeline replication	18
Insufficient metadata	9
Qualified	0

Table S8: Software and environment versions recorded for the frozen analysis. Versioned configuration records and analysis outputs provide the reproducibility record for the reported analyses.

Software	Version	Runtime role
Python	3.12.13	runtime
NumPy	2.5.1	numeric arrays
pandas	3.0.5	tabular data
SciPy	1.18.0	scientific routines
scikit-learn	1.9.0	preprocessing and linear models
LightGBM	4.7.0	boosted-tree model
PyYAML	6.0.3	configuration and protocol parsing
matplotlib	3.11.1	figure generation
crc-trustbench	0.1.0	local audited analysis code
Analysis protocol	2026-08-02	frozen LOCO, calibration, conformal, selective and shift-analysis configuration

5 Additional sensitivity analyses and audit summaries

5.1 Repeated RANDOM sensitivity and domain composition

Thirty repeated subject-aware RANDOM splits were evaluated for M0 Elastic Net and M1 LightGBM using fixed split seeds 2026083101–2026083130. Population eligibility, the 70/10/10/10 training, validation, calibration, and test role proportions, study-by-diagnosis stratification, fold-local preprocessing, validation-only tuning, and the model seed were held fixed. Every test role contained 205 samples (93 CRC and 112 healthy). Supplementary Table S9 reports the split-level distribution summaries. This sensitivity analysis quantifies split-to-split variability; it does not replace the single prespecified RANDOM split, was not treated as a fourth primary validation protocol, and was not used for significance comparisons among validation protocols.

The complete study-by-country cross-tabulation comprises 15 studies and 10 countries (Supplementary Table S10). Thirteen studies were represented in one country, whereas PRJEB6070 and PRJNA389927 each spanned two countries. Consequently, LOCO evaluation does not necessarily remove every study-associated processing characteristic when a country is held out. Supplementary Table S11 gives the class composition of all 15 LOSO and all 10 LOCO test folds. The two single-class LOSO folds were retained in the analysis record, while AUROC and AUPRC were reported as NA for those folds rather than assigned 0 or 0.5. All 10 LOCO folds contained both classes; CAN contained only three samples (one CRC and two healthy). The prespecified primary LOCO summaries retained all 10 countries, and Supplementary Table S17 reports the descriptive excluding-CAN re-aggregation without model retraining.

5.2 Calibration and domain-level uncertainty

Primary calibration roles contained 140–212 records per country and model. In the strict sensitivity, subject-disjoint Calibration-A and Calibration-B roles contained 70–106 records each; Calibration-A was used for Platt scaling and Calibration-B for conformity-score quantiles (Supplementary Table S12). The domain-macro primary and strict results for M0, M1, and M2 at nominal 90% and 95% coverage are reported in Supplementary Table S13. These are empirical conformal-style summaries. Reuse of the primary calibration role and possible non-exchangeability of held-out countries preclude interpretation as a finite-sample coverage guarantee for future populations.

Supplementary Table S14 reports exploratory percentile bootstrap intervals based on 10,000 resamples with seed 2026083199. The resampling unit was the held-out study for LOSO and the held-out country for LOCO. These intervals summarize between-domain uncertainty among the observed studies or countries; they are neither prediction intervals for future domains nor clinical confidence intervals.

5.3 MiLCA method qualification

The feature-level MiLCA audit used 1,953 Task 1 subject groups and 2,543 fixed-pseudocount centered-log-ratio taxonomic features. Nested least-squares designs comprised an intercept-only model, diagnosis alone, study alone, and diagnosis plus study. The study term contained 14 indicators for 15 studies, with PRJDB4176 as the reference, whereas diagnosis was one binary indicator. Let $\mathrm{RSS}_{\mathrm{full}}$ denote the residual sum of squares from the joint model containing an intercept, diagnosis, and study, and let $\mathrm{RSS}_{\mathrm{reduced}}$ denote the residual sum of squares after removing the term being evaluated. Partial R^2 was defined as

$$R^2_{\mathrm{partial}} = \frac{\mathrm{RSS}_{\mathrm{reduced}} - \mathrm{RSS}_{\mathrm{full}}}{\mathrm{RSS}_{\mathrm{reduced}}}.$$

For the diagnosis-associated effect, the reduced model contained an intercept and study; for the study-associated effect, the reduced model contained an intercept and diagnosis. Diagnosis-associated effects were therefore adjusted for study, and study-associated effects were adjusted for diagnosis. Age, sex, body mass index, treatment, stage, platform, and other clinical covariates were not included. The fixed population required non-empty study and diagnosis labels, invalid abundance rows were rejected, no missing-value imputation was applied, and all 1,953 rows and 2,543 features were retained.

The comparison criterion was the strict feature-wise direction $R^2_{\mathrm{study}} > R^2_{\mathrm{diagnosis}}$, with ties recorded separately. No feature-wise significance tests or p values were generated, so no multiplicity correction was applied. The finding that 99.21% of features met this directional criterion is descriptive, associational, and not complexity-adjusted. Because the study and diagnosis terms differ substantially in dimensionality, the result does not establish causal attribution, statistical significance, or dominance of one source of variation over the other.

5.4 Clinical metadata and external-resource audit

Supplementary Table S15 reports the recorded availability of source class labels, diagnostic confirmation, stage, treatment status, age, and sex for all 15 studies. Only observed fields and missingness counts are shown; no unavailable clinical fields were imputed and no additional models were fitted. Supplementary Table S16 lists all 58 frozen external project-level records screened for CRC relevance, sample-level independence, authorization, and compatibility with the frozen feature space. None met all eligibility criteria. This inventory provides an auditable qualification boundary and is not an independent validation analysis.

5.5 Stable candidate terminology

Throughout the interpretation of the study's own selected features, the taxonomic and functional tiers denote stable candidate hypotheses rather than validated biomarkers or mechanisms. No independent cohort, raw-data reprocessing, or experimental validation was available. Because the

taxonomic and functional feature spaces differ in dimensionality and correlation structure, their tier counts cannot be interpreted as evidence of statistical or biological dominance.

Table S9: Repeated subject-aware RANDOM sensitivity for M0 Elastic Net and M1 LightGBM across 30 fixed split seeds. Each test role contained 205 samples (93 CRC and 112 healthy). Values summarize split-level test AUROC, AUPRC, and Brier score; higher AUROC and AUPRC and lower Brier score indicate better performance. SD is the sample standard deviation, IQR gives the 25th–75th percentiles, and P2.5–P97.5 gives the empirical 2.5th–97.5th percentiles. The analysis quantifies split-to-split variability and does not replace the primary prespecified RANDOM result.

Model	Metric	Mean	Median	SD	IQR	P2.5–P97.5
M0 Elastic Net	AUROC	0.842	0.844	0.031	0.828–0.863	0.787–0.888
M0 Elastic Net	AUPRC	0.838	0.842	0.032	0.817–0.859	0.775–0.881
M0 Elastic Net	Brier score	0.159	0.159	0.017	0.147–0.167	0.135–0.190
M1 LightGBM	AUROC	0.857	0.856	0.027	0.841–0.872	0.808–0.904
M1 LightGBM	AUPRC	0.851	0.849	0.025	0.836–0.868	0.803–0.896
M1 LightGBM	Brier score	0.162	0.162	0.018	0.150–0.172	0.125–0.192

Table S10: Study-by-country composition of the frozen Task 1 CRC-versus-healthy population (1,953 samples, 15 studies, and 10 countries). Counts are sample counts. A multi-country study is represented in more than one country. LOCO denotes leave-one-country-out evaluation; multi-country studies mean that holding out a country does not necessarily remove every study-associated processing characteristic.

Study	Country	CRC, n	Healthy, n	Total, n	Multi-country study
PRJDB4176	JPN	257	290	547	NO
PRJEB10878	CHN	70	53	123	NO
PRJEB12449	USA	50	52	102	NO
PRJEB27928	DEU	21	58	79	NO
PRJEB6070	DEU	35	5	40	YES
PRJEB6070	FRA	52	60	112	YES
PRJEB7774	AUT	34	63	97	NO
PRJNA389927	CAN	1	2	3	YES
PRJNA389927	USA	14	13	27	YES
PRJNA397112	IND	0	74	74	NO
PRJNA429097	CHN	96	93	189	NO
PRJNA447983	ITA	57	48	105	NO
PRJNA531273	IND	11	0	11	NO
PRJNA608088	CHN	6	12	18	NO
PRJNA731589	CHN	68	76	144	NO
PRJNA763023	CHN	95	97	192	NO
PRJNA961076	BRA	30	60	90	NO

Table S11: Class composition and metric evaluability for every leave-one-study-out (LOSO) and leave-one-country-out (LOCO) test fold in the frozen Task 1 population. AUROC and AUPRC are not estimable for single-class test folds and are not assigned 0 or 0.5. CRC denotes colorectal cancer; NONE indicates no non-estimability reason.

Protocol	Held-out domain	CRC, n	Healthy, n	Total, n	AUROC evaluable	AUPRC evaluable	Non-estimability reason
LOSO	PRJDB4176	257	290	547	YES	YES	NONE
LOSO	PRJEB10878	70	53	123	YES	YES	NONE
LOSO	PRJEB12449	50	52	102	YES	YES	NONE
LOSO	PRJEB27928	21	58	79	YES	YES	NONE
LOSO	PRJEB6070	87	65	152	YES	YES	NONE
LOSO	PRJEB7774	34	63	97	YES	YES	NONE
LOSO	PRJNA389927	15	15	30	YES	YES	NONE
LOSO	PRJNA397112	0	74	74	NO	NO	SINGLE_CLASS_TEST_FOLD_MISSING_CRC
LOSO	PRJNA429097	96	93	189	YES	YES	NONE
LOSO	PRJNA447983	57	48	105	YES	YES	NONE
LOSO	PRJNA531273	11	0	11	NO	NO	SINGLE_CLASS_TEST_FOLD_MISSING_HEALTHY
LOSO	PRJNA608088	6	12	18	YES	YES	NONE
LOSO	PRJNA731589	68	76	144	YES	YES	NONE
LOSO	PRJNA763023	95	97	192	YES	YES	NONE
LOSO	PRJNA961076	30	60	90	YES	YES	NONE
LOCO	AUT	34	63	97	YES	YES	NONE
LOCO	BRA	30	60	90	YES	YES	NONE
LOCO	CAN	1	2	3	YES	YES	NONE
LOCO	CHN	335	331	666	YES	YES	NONE
LOCO	DEU	56	63	119	YES	YES	NONE
LOCO	FRA	52	60	112	YES	YES	NONE
LOCO	IND	11	74	85	YES	YES	NONE
LOCO	ITA	57	48	105	YES	YES	NONE
LOCO	JPN	257	290	547	YES	YES	NONE
LOCO	USA	64	65	129	YES	YES	NONE

Table S12: Calibration-record counts by held-out country and model for the primary LOCO trust layer and the strict Calibration-A/Calibration-B sensitivity. Calibration-A fits Platt scaling, Calibration-B supplies conformal-style conformity scores and quantiles, and the held-out country remains evaluation-only. M0 is Elastic Net, M1 is LightGBM, and M2 is cohort-balanced Elastic Net; all strict role-construction checks passed.

Country	Model	Full calibration, n	Calibration-A, n	Calibration-B, n	Status
AUT	M0 Elastic Net	201	101	100	PASS
AUT	M1 LightGBM	201	101	100	PASS
AUT	M2 cohort-balanced Elastic Net	201	101	100	PASS
BRA	M0 Elastic Net	201	101	100	PASS
BRA	M1 LightGBM	201	101	100	PASS
BRA	M2 cohort-balanced Elastic Net	201	101	100	PASS
CAN	M0 Elastic Net	212	106	106	PASS
CAN	M1 LightGBM	212	106	106	PASS
CAN	M2 cohort-balanced Elastic Net	212	106	106	PASS
CHN	M0 Elastic Net	140	70	70	PASS
CHN	M1 LightGBM	140	70	70	PASS
CHN	M2 cohort-balanced Elastic Net	140	70	70	PASS
DEU	M0 Elastic Net	200	100	100	PASS
DEU	M1 LightGBM	200	100	100	PASS
DEU	M2 cohort-balanced Elastic Net	200	100	100	PASS
FRA	M0 Elastic Net	201	101	100	PASS
FRA	M1 LightGBM	201	101	100	PASS
FRA	M2 cohort-balanced Elastic Net	201	101	100	PASS
IND	M0 Elastic Net	202	101	101	PASS
IND	M1 LightGBM	202	101	101	PASS
IND	M2 cohort-balanced Elastic Net	202	101	101	PASS
ITA	M0 Elastic Net	201	101	100	PASS
ITA	M1 LightGBM	201	101	100	PASS
ITA	M2 cohort-balanced Elastic Net	201	101	100	PASS
JPN	M0 Elastic Net	156	78	78	PASS
JPN	M1 LightGBM	156	78	78	PASS
JPN	M2 cohort-balanced Elastic Net	156	78	78	PASS
USA	M0 Elastic Net	196	98	98	PASS
USA	M1 LightGBM	196	98	98	PASS
USA	M2 cohort-balanced Elastic Net	196	98	98	PASS

Table S13: Domain-macro comparison across 10 held-out countries of the primary same-calibration conformal-style procedure and the frozen strict Calibration-A/Calibration-B sensitivity. M0 is Elastic Net, M1 is LightGBM, and M2 is cohort-balanced Elastic Net. Set size is the mean prediction-set size; coverage is interpreted relative to the nominal target, and lower Brier score and log loss indicate better probability accuracy. These empirical summaries do not provide a finite-sample guarantee for future populations.

Model	Nominal	Primary coverage	Strict coverage	Primary set size	Strict set size	Primary Brier	Strict Brier	Primary log loss	Strict log loss
M0 Elastic Net	0.900	0.888	0.878	1.377	1.363	0.186	0.184	0.562	0.553
M0 Elastic Net	0.950	0.936	0.918	1.608	1.611	0.186	0.184	0.562	0.553
M1 LightGBM	0.900	0.869	0.863	1.267	1.256	0.169	0.171	0.521	0.525
M1 LightGBM	0.950	0.931	0.937	1.512	1.560	0.169	0.171	0.521	0.525
M2 cohort-balanced Elastic Net	0.900	0.890	0.880	1.395	1.390	0.175	0.175	0.534	0.529
M2 cohort-balanced Elastic Net	0.950	0.945	0.944	1.604	1.616	0.175	0.175	0.534	0.529

Table S14: Exploratory between-domain uncertainty for frozen discrimination, calibrated Brier score, and empirical conformal-style coverage. LOSO resampled held-out studies and LOCO resampled held-out countries using 10,000 percentile bootstrap replicates with seed 2026083199. M0 is Elastic Net, M1 is LightGBM, and M2 is cohort-balanced Elastic Net. NA denotes no operating point for the metric. Higher AUROC and AUPRC and lower Brier score indicate better performance; coverage is interpreted relative to its nominal target. Intervals summarize the observed domains and are not future-domain prediction intervals or clinical confidence intervals.

Protocol	Analysis	Model	Metric	Operating point	Domain-macro mean	Bootstrap 95% interval	Domains, n
LOCO	Discrimination	M0 Elastic Net	AUPRC	NA	0.774	0.701–0.847	10
LOCO	Discrimination	M0 Elastic Net	AUROC	NA	0.782	0.718–0.851	10
LOCO	Discrimination	M1 LightGBM	AUPRC	NA	0.803	0.734–0.869	10
LOCO	Discrimination	M1 LightGBM	AUROC	NA	0.821	0.773–0.877	10
LOCO	Discrimination	M2 cohort-balanced Elastic Net	AUPRC	NA	0.798	0.740–0.859	10
LOCO	Discrimination	M2 cohort-balanced Elastic Net	AUROC	NA	0.811	0.744–0.875	10
LOCO	Platt calibration	M0 Elastic Net	Brier score	NA	0.186	0.145–0.224	10
LOCO	Platt calibration	M1 LightGBM	Brier score	NA	0.169	0.132–0.200	10
LOCO	Platt calibration	M2 cohort-balanced Elastic Net	Brier score	NA	0.175	0.138–0.212	10
LOCO	Conformal-style	M0 Elastic Net	Coverage	Nominal 0.90	0.888	0.832–0.936	10
LOCO	Conformal-style	M0 Elastic Net	Coverage	Nominal 0.95	0.936	0.904–0.965	10
LOCO	Conformal-style	M1 LightGBM	Coverage	Nominal 0.90	0.869	0.817–0.920	10
LOCO	Conformal-style	M1 LightGBM	Coverage	Nominal 0.95	0.931	0.901–0.963	10
LOCO	Conformal-style	M2 cohort-balanced Elastic Net	Coverage	Nominal 0.90	0.890	0.845–0.936	10
LOCO	Conformal-style	M2 cohort-balanced Elastic Net	Coverage	Nominal 0.95	0.945	0.917–0.972	10
LOSO	Discrimination	M0 Elastic Net	AUPRC	NA	0.812	0.759–0.867	13
LOSO	Discrimination	M0 Elastic Net	AUROC	NA	0.809	0.753–0.863	13
LOSO	Discrimination	M1 LightGBM	AUPRC	NA	0.834	0.786–0.882	13
LOSO	Discrimination	M1 LightGBM	AUROC	NA	0.827	0.782–0.874	13
LOSO	Discrimination	M2 cohort-balanced Elastic Net	AUPRC	NA	0.818	0.764–0.873	13
LOSO	Discrimination	M2 cohort-balanced Elastic Net	AUROC	NA	0.816	0.766–0.869	13

Table S15: Availability of recorded clinical metadata for all 15 Task 1 studies. CRC and healthy definition columns use SL for an available explicit source class label, NA-C for no CRC samples, and NA-H for no healthy samples. Diagnostic confirmation, stage, and treatment use U for unavailable in the canonical records. Age and sex use C, P, and U for complete, partial, and unavailable, with nonmissing counts in parentheses. NA denotes unavailable. Sex counts are female/male/other or unknown. Only observed fields and missingness are reported; no unavailable value was imputed and no additional model was fitted.

Study	CRC definition	Healthy definition	Diagnostic confirmation	Stage	Treatment	Age status (n)	Sex status (n)	Age median	Age IQR	Sex counts
PRJDB4176	SL	SL	U	U	U	C (547)	C (547)	64.000	54.5–70.0	239/308/0
PRJEB10878	SL	SL	U	U	U	C (123)	C (123)	64.000	58.5–70.0	46/77/0
PRJEB12449	SL	SL	U	U	U	C (102)	C (102)	63.000	54.0–70.0	29/73/0
PRJEB27928	SL	SL	U	U	U	C (79)	C (79)	60.000	53.0–65.5	38/41/0
PRJEB6070	SL	SL	U	U	U	C (152)	C (152)	63.000	55.0–70.0	67/85/0
PRJEB7774	SL	SL	U	U	U	C (97)	C (97)	68.000	64.0–72.0	38/59/0
PRJNA389927	SL	SL	U	U	U	C (30)	C (30)	55.500	51.0–62.5	15/15/0
PRJNA397112	NA-C	SL	U	U	U	C (74)	C (74)	27.000	24.0–30.0	37/37/0
PRJNA429097	SL	SL	U	U	U	C (189)	C (189)	48.000	41.0–59.0	76/113/0
PRJNA447983	SL	SL	U	U	U	C (105)	C (105)	64.000	58.0–69.0	36/69/0
PRJNA531273	SL	NA-H	U	U	U	C (11)	C (11)	59.000	54.0–68.0	5/6/0
PRJNA608088	SL	SL	U	U	U	U (0)	P (6)	NA	NA	0/6/12
PRJNA731589	SL	SL	U	U	U	C (144)	P (143)	60.000	53.0–65.0	58/85/1
PRJNA763023	SL	SL	U	U	U	U (0)	U (0)	NA	NA	0/0/192
PRJNA961076	SL	SL	U	U	U	C (90)	C (90)	62.000	54.0–69.8	44/46/0

Table S16: Complete audit of 58 frozen project-level external-resource records. Formal eligibility required CRC relevance, sample-level independence, authorized use, and compatibility with the frozen feature space simultaneously. cMD is curatedMetagenomicData. CRC relevance codes are NR, not relevant; RP, CRC-relevant pipeline replication; and UIM, unresolved because of insufficient metadata. Sample-identity codes are NR, not relevant; PR, pipeline replication; and IM, insufficient metadata. Independence codes are NA, not applicable; NI, not independent; and NE, not established. UNK is unknown, NE in the compatibility column is not evaluated, and INEL is ineligible. Exclusion codes are A, not a primary human stool shotgun resource and license unverified; B, pipeline replication not independent and license unverified; and C, sample-level identity insufficient and license unverified. No resource was eligible, and no new resource search or independent validation analysis was performed.

Resource	Project	CRC relevance	Sample identity	Independence	Authorization	Compatibility	Eligibility	Exclusion
GMrepo_v3	PRJDB11246	NR	NR	NA	UNK	NE	INEL	A
GMrepo_v3	PRJDB11845	NR	NR	NA	UNK	NE	INEL	A
GMrepo_v3	PRJEB13249	NR	NR	NA	UNK	NE	INEL	A
GMrepo_v3	PRJEB35990	NR	NR	NA	UNK	NE	INEL	A
GMrepo_v3	PRJEB46665	NR	NR	NA	UNK	NE	INEL	A
GMrepo_v3	PRJEB53415	NR	NR	NA	UNK	NE	INEL	A
GMrepo_v3	PRJEB53891	NR	NR	NA	UNK	NE	INEL	A
GMrepo_v3	PRJEB70916	UIM	IM	NE	UNK	NE	INEL	C
GMrepo_v3	PRJNA1018228	NR	NR	NA	UNK	NE	INEL	A
GMrepo_v3	PRJNA1022861	NR	NR	NA	UNK	NE	INEL	A
GMrepo_v3	PRJNA1050885	UIM	IM	NE	UNK	NE	INEL	C
GMrepo_v3	PRJNA1126038	NR	NR	NA	UNK	NE	INEL	A
GMrepo_v3	PRJNA1138893	UIM	IM	NE	UNK	NE	INEL	C
GMrepo_v3	PRJNA1167935	UIM	IM	NE	UNK	NE	INEL	C
GMrepo_v3	PRJNA243409	NR	NR	NA	UNK	NE	INEL	A
GMrepo_v3	PRJNA284542	NR	NR	NA	UNK	NE	INEL	A
GMrepo_v3	PRJNA325649	NR	NR	NA	UNK	NE	INEL	A
GMrepo_v3	PRJNA325650	NR	NR	NA	UNK	NE	INEL	A
GMrepo_v3	PRJNA397112	RP	PR	NI	UNK	NE	INEL	B
GMrepo_v3	PRJNA429097	RP	PR	NI	UNK	NE	INEL	B
GMrepo_v3	PRJNA449268	NR	NR	NA	UNK	NE	INEL	A
GMrepo_v3	PRJNA464414	NR	NR	NA	UNK	NE	INEL	A
GMrepo_v3	PRJNA478491	NR	NR	NA	UNK	NE	INEL	A
GMrepo_v3	PRJNA511474	NR	NR	NA	UNK	NE	INEL	A
GMrepo_v3	PRJNA531273	RP	PR	NI	UNK	NE	INEL	B
GMrepo_v3	PRJNA599023	NR	NR	NA	UNK	NE	INEL	A
GMrepo_v3	PRJNA692325	UIM	IM	NE	UNK	NE	INEL	C
GMrepo_v3	PRJNA698923	NR	NR	NA	UNK	NE	INEL	A
GMrepo_v3	PRJNA731589	RP	PR	NI	UNK	NE	INEL	B
GMrepo_v3	PRJNA754518	UIM	IM	NE	UNK	NE	INEL	C
GMrepo_v3	PRJNA763023	RP	PR	NI	UNK	NE	INEL	B
GMrepo_v3	PRJNA797640	NR	NR	NA	UNK	NE	INEL	A
GMrepo_v3	PRJNA824020	NR	NR	NA	UNK	NE	INEL	A
GMrepo_v3	PRJNA825696	NR	NR	NA	UNK	NE	INEL	A
GMrepo_v3	PRJNA846495	UIM	IM	NE	UNK	NE	INEL	C
GMrepo_v3	PRJNA859426	NR	NR	NA	UNK	NE	INEL	A
GMrepo_v3	PRJNA869306	NR	NR	NA	UNK	NE	INEL	A
GMrepo_v3	PRJNA869338	NR	NR	NA	UNK	NE	INEL	A
GMrepo_v3	PRJNA888860	UIM	IM	NE	UNK	NE	INEL	C
GMrepo_v3	PRJNA904946	NR	NR	NA	UNK	NE	INEL	A
GMrepo_v3	PRJNA936589	UIM	IM	NE	UNK	NE	INEL	C
GMrepo_v3	PRJNA954195	NR	NR	NA	UNK	NE	INEL	A
GMrepo_v3	PRJNA961076	RP	PR	NI	UNK	NE	INEL	B
GMrepo_v3	PRJNA986115	NR	NR	NA	UNK	NE	INEL	A
GMrepo_v3	PRJNA986175	NR	NR	NA	UNK	NE	INEL	A
GMrepo_v3	PRJNA989099	NR	NR	NA	UNK	NE	INEL	A
cMD	DhakanDB_2019	RP	PR	NI	UNK	NE	INEL	B
cMD	FengQ_2015	RP	PR	NI	UNK	NE	INEL	B
cMD	GuptaA_2019	RP	PR	NI	UNK	NE	INEL	B
cMD	HanniganGD_2017	RP	PR	NI	UNK	NE	INEL	B
cMD	ThomasAM_2018a	RP	PR	NI	UNK	NE	INEL	B
cMD	ThomasAM_2018b	RP	PR	NI	UNK	NE	INEL	B
cMD	ThomasAM_2019_c	RP	PR	NI	UNK	NE	INEL	B
cMD	VogtmannE_2016	RP	PR	NI	UNK	NE	INEL	B
cMD	WirbelJ_2018	RP	PR	NI	UNK	NE	INEL	B
cMD	YachidaS_2019	RP	PR	NI	UNK	NE	INEL	B
cMD	YuJ_2015	RP	PR	NI	UNK	NE	INEL	B
cMD	ZellerG_2014	RP	PR	NI	UNK	NE	INEL	B

Table S17: LOCO sensitivity analysis with and without the CAN fold. With CAN is the prespecified primary analysis across ten countries; without CAN is a descriptive re-aggregation across nine countries. Country-level results were frozen, macro summaries are unweighted across evaluable countries, and no model was retrained. CAN contained three samples (one CRC and two healthy). Tax. and Func. denote taxonomic and functional profiles. M0 is Elastic Net, M1 is LightGBM, and M2 is cohort-balanced Elastic Net. Values and absolute differences are displayed to three decimals after calculation at full available precision.

Domain	Model	Metric and operating point	With CAN	Without CAN	$\lvert\Delta\rvert$
Tax.	M0	AUROC	0.782	0.758	0.024
Tax.	M0	AUPRC	0.774	0.749	0.025
Tax.	M0	Raw Brier	0.198	0.218	0.020
Tax.	M0	Raw log loss	0.644	0.701	0.058
Tax.	M0	Platt Brier	0.186	0.201	0.015
Tax.	M0	Platt log loss	0.562	0.597	0.035
Tax.	M0	Conformal-style coverage, 90%	0.888	0.876	0.012
Tax.	M0	Mean set size, 90%	1.377	1.419	0.042
Tax.	M0	Conformal-style coverage, 95%	0.936	0.929	0.007
Tax.	M0	Mean set size, 95%	1.608	1.601	0.007
Tax.	M0	Selective retained coverage, 80%	0.801	0.779	0.022
Tax.	M0	Selective error, 80%	0.230	0.255	0.026
Tax.	M0	Selective retained coverage, 90%	0.900	0.889	0.011
Tax.	M0	Selective error, 90%	0.247	0.274	0.027
Tax.	M0	CRC–healthy retention gap, 80%	0.054	0.060	0.006
Tax.	M0	Better-than-random fraction, 80%	0.852	0.946	0.095
Func.	M0	AUROC	0.690	0.712	0.021
Tax.	M1	AUROC	0.821	0.801	0.020
Tax.	M1	AUPRC	0.803	0.781	0.022
Tax.	M1	Raw Brier	0.180	0.199	0.019
Tax.	M1	Raw log loss	0.630	0.690	0.060
Tax.	M1	Platt Brier	0.169	0.183	0.014
Tax.	M1	Platt log loss	0.521	0.555	0.034
Tax.	M1	Conformal-style coverage, 90%	0.869	0.854	0.015
Tax.	M1	Mean set size, 90%	1.267	1.297	0.030
Tax.	M1	Conformal-style coverage, 95%	0.931	0.924	0.008
Tax.	M1	Mean set size, 95%	1.512	1.532	0.020
Tax.	M1	Selective retained coverage, 80%	0.814	0.793	0.021
Tax.	M1	Selective error, 80%	0.207	0.230	0.023
Tax.	M1	Selective retained coverage, 90%	0.905	0.894	0.011
Tax.	M1	Selective error, 90%	0.220	0.245	0.024
Tax.	M1	CRC–healthy retention gap, 80%	0.010	0.011	0.001
Tax.	M1	Better-than-random fraction, 80%	0.812	0.902	0.090
Tax.	M1	Better-than-random fraction, 90%	0.754	0.837	0.084
Func.	M1	AUROC	0.743	0.714	0.029

Continued on next page

Table S17 (continued)

Domain	Model	Metric and operating point	With CAN	Without CAN	$\lvert\Delta\rvert$
Tax.	M2	AUROC	0.811	0.790	0.021
Tax.	M2	AUPRC	0.798	0.775	0.022
Tax.	M2	Raw Brier	0.190	0.201	0.011
Tax.	M2	Raw log loss	0.609	0.639	0.030
Tax.	M2	Platt Brier	0.175	0.190	0.014
Tax.	M2	Platt log loss	0.534	0.570	0.036
Tax.	M2	Conformal-style coverage, 90%	0.890	0.878	0.012
Tax.	M2	Mean set size, 90%	1.395	1.402	0.007
Tax.	M2	Conformal-style coverage, 95%	0.945	0.939	0.006
Tax.	M2	Mean set size, 95%	1.604	1.634	0.030
Tax.	M2	Selective retained coverage, 80%	0.793	0.770	0.023
Tax.	M2	Selective error, 80%	0.196	0.218	0.022
Tax.	M2	Selective retained coverage, 90%	0.914	0.904	0.010
Tax.	M2	Selective error, 90%	0.229	0.255	0.025
Tax.	M2	CRC–healthy retention gap, 80%	0.017	0.019	0.002
Tax.	M2	Better-than-random fraction, 80%	0.784	0.872	0.087
Func.	M2	AUROC	0.626	0.696	0.070
Tax.	M1	Strict A/B coverage, 90%	0.863	0.848	0.015
Tax.	M1	Strict A/B Brier	0.171	0.185	0.014
Tax.	M1	Strict A/B log loss	0.525	0.557	0.032
Tax.	M1	Strict A/B coverage, 95%	0.937	0.930	0.007
Tax.	M0	Worst-country AUROC (USA)	0.627	0.627	0.000
Tax.	M1	Worst-country AUROC (JPN)	0.697	0.697	0.000
Tax.	M2	Worst-country AUROC (JPN)	0.648	0.648	0.000

Conformal-style percentages indicate nominal coverage; selective and matched-random percentages indicate target retained coverage. Strict A/B denotes the frozen subject-disjoint Calibration-A/Calibration-B sensitivity, summarized separately from the primary same-calibration procedure. Worst-country AUROC is the minimum across evaluable countries, with the country shown in parentheses. Retention gap is the absolute difference between the unrounded country-macro CRC and healthy retention rates, not the mean of country-specific absolute gaps. Better-than-random is the country-macro mean of the frozen fractions of 200 matched-random repeats with higher error than uncertainty-based abstention. Higher AUROC and AUPRC and lower Brier score, log loss, and selective error indicate better performance. Coverage remains an empirical summary. No without-CAN bootstrap interval was generated; the M1 LOCO interval in Supplementary Table S14 applies only to the primary ten-country analysis. Full-precision summaries and source-file references are provided in `Supplementary_Data/loco_can_sensitivity.tsv`.
